# Completeness and quality of comprehensive managed care data compared with fee‐for‐service data in national Medicaid claims from 2001 to 2019

**DOI:** 10.1111/1475-6773.14429

**Published:** 2025-01-02

**Authors:** Hillary Samples, Kristen Lloyd, Radha Ryali, Silvia S. Martins, Magdalena Cerdá, Deborah Hasin, Stephen Crystal, Mark Olfson

**Affiliations:** ^1^ Institute for Health, Health Care Policy and Aging Research Rutgers University New Brunswick New Jersey USA; ^2^ Department of Health Behavior, Society and Policy Rutgers School of Public Health Piscataway New Jersey USA; ^3^ Department of Epidemiology Columbia University Mailman School of Public Health New York New York USA; ^4^ Department of Population Health NYU Grossman School of Medicine New York New York USA; ^5^ Department of Psychiatry, New York State Psychiatric Institute Columbia University Irving Medical Center New York New York USA

**Keywords:** data accuracy, data quality, fee‐for‐service plans, managed care programs, Medicaid

## Abstract

**Objective:**

To evaluate the completeness and quality of Medicaid comprehensive managed care (CMC) data in national MAX/TAF research files.

**Study Setting and Design:**

This observational study compared CMC with fee‐for‐service (FFS) enrollee data in 2001–2019 Medicaid MAX/TAF inpatient, outpatient, and pharmacy files. Completeness was assessed as the proportion of enrollees with any claim and mean claims per enrollee with any claim. Quality was assessed as the proportion of inpatient and outpatient claims with primary diagnosis and procedure codes and the proportion of prescription drug claims with fill dates, National Drug Codes (NDC), days supplied, and quantity dispensed. Acceptable ranges for each study measure were defined as the national FFS mean ± 2 standard deviations.

**Data Sources and Analytic Sample:**

We analyzed secondary data on 45 states from 2001 to 2013 (MAX) and 50 states and DC from 2014 to 2019 (TAF). The sample included adults aged 18–64 with continuous calendar‐year enrollment who were eligible for full Medicaid benefits and ineligible for Medicare. We determined CMC enrollment rates and assessed data completeness and quality among state‐years with ≥10% CMC penetration, comparing CMC with FFS enrollees.

**Principal Findings:**

Across 891 state‐years, 194,364,647 enrollees met inclusion criteria. Of 540 state‐years (60.6%) with ≥10% CMC enrollment, CMC data were largely comparable to national FFS distributions for all inpatient (*n* = 430; 79.6%), outpatient (*n* = 467, 86.5%), and prescription (*n* = 459, 85.0%) completeness criteria and for all inpatient (*n* = 449, 83.1%), outpatient (*n* = 511, 94.6%), and prescription (*n* = 528, 97.8%) quality criteria. Overall completeness (92.3%) and quality (84.6%) improved substantially by 2019.

**Conclusions:**

Completeness and quality of CMC data were largely comparable to FFS data, with increasing state‐years meeting criteria over time. Further research on national Medicaid populations should assess and address differences in data completeness and quality by plan type across states, over time, and in relation to specific study samples and measures of interest.

**What is known on this topic**

- Medicaid managed care encounter data are conceptually similar to fee‐for‐service (FFS) claims data, documenting enrollee service use covered by managed care organizations.
- Managed care data are more complex to collect and report, contributing to concerns over data completeness and quality that may lead to excluding managed care populations from research.
- Evidence on managed care data completeness and quality to support research using national Medicaid data is outdated and limited to select years.

**What this study adds**

- In a systematic study of national US Medicaid research files from 2001 to 2019, data for comprehensive managed care (CMC) enrollees were largely comparable to data for fee‐for‐service (FFS) enrollees.
- Beginning in 2010, >80% of states met the criteria for low‐concern CMC data across all measures of completeness and all measures of quality in inpatient, outpatient, and pharmacy data files.
- Modest declines in the comparability of CMC and FFS data completeness and quality coincided with state transitions to a new reporting system (2014–2016) and began to rebound thereafter (2017–2019).

## 1 INTRODUCTION

Medicaid covers approximately 80 million low‐income Americans, including over 40 million adults 18–64 years old, who represent a high‐risk population for adverse health outcomes and a high‐need population for health services. To facilitate evidence‐based advances in health policies and programs for this population, the Centers for Medicare & Medicaid Services (CMS) provide access to research files containing national Medicaid data. These files are a critical data source for academic health services and policy research and for government reports.^1^, ^2^, ^3^, ^4^, ^5^, ^6^, ^7^, ^8^ They include enrollment information and health care claims and encounter data submitted to CMS by each state program. Although data reporting has been required by federal law since 1999,^9^ requirements have expanded in recent years to ensure the submission of complete, accurate, and timely data, specifically for managed care organizations.^10^, ^11^

States may provide Medicaid benefits through fee‐for‐service (FFS) or managed care plans (or both). While managed care data are conceptually equivalent to FFS data, managed care encounters are more complicated in terms of data collection and reporting. For example, while each health service is billed and reimbursed directly by FFS plans, managed care plans may pay providers fixed amounts on a regular basis. Because information required for reimbursement is generally higher quality,^12^ FFS data may be higher quality. Moreover, high‐quality managed care data depend on strong communication and coordination across multiple entities. Because managed care reflects multiple payers instead of a single payer (the state), there may be differences across managed care organizations in filing requirements, such as the level of detail about services delivered. In addition, multiple Medicaid program staff may be responsible for data processing, including information technology staff, managed care operations and policy staff, and claims processors. Further, agency staff must often coordinate with external actuaries, review organizations, and fiscal agents to assess data quality.

To address concerns about the completeness and quality of managed care encounter data, CMS previously commissioned assessments of the national Medicaid research files.^13^, ^14^, ^15^, ^16^ Comparing comprehensive managed care (CMC) with FFS data from 2007 to 2011, availability, completeness, and quality of CMC data were generally comparable to FFS claims, with some indications of improvement over time.^13^, ^14^, ^15^, ^16^ A separate analysis on a subset of states from 2007 to 2010 used different quality indicators but similarly found that CMC data quality was stable or improving over time.^17^

Concerns about managed care data, however, rose alongside growth in Medicaid managed care enrollment. While managed care penetration varies by state, most states (41 in 2021) now have private CMC contracts covering all or a portion of their enrolled population.^18^ Nationwide, more than two thirds of Medicaid beneficiaries are currently enrolled in CMC plans (70.1% in 2019),^19^ underscoring the importance of evaluating the completeness and quality of these data for research. While health services and policy studies commonly focus on FFS enrollees due to concerns about data integrity,^20^, ^21^, ^22^, ^23^ excluding managed care enrollees can severely, and increasingly, limit the generalizability of findings to Medicaid populations.

Little is known about the quality of more recent encounter data after the enrolled population substantially increased because of Medicaid expansion. By the end of September 2021, the 38 states that adopted Medicaid expansion encompassed over 16 million newly eligible enrollees.^24^ Additionally, there has been a change in the system states use to submit data to CMS and the format of research files created from state submissions. Until 2011, states submitted data using the Medicaid Statistical Information System (MSIS), transitioning to the Transformed MSIS (T‐MSIS) over the next 5 years. In 2014 and 2015, as the last states shifted to T‐MSIS, research files transitioned from the Medicaid Analytic eXtract (MAX) format to the T‐MSIS Analytic Files (TAF) format.^25^

Considering recent policy and technology advancements to strengthen data reporting alongside changes in Medicaid programs and populations, addressing gaps in evidence about the completeness and quality of CMC data over time is critical to inform health services research focused on Medicaid. Using data acquired from CMS, the objective of this study was to identify states with CMC data comparable to data for the FFS population over an extended period (2001–2019). To accomplish this, we analyzed inpatient, outpatient, and pharmacy files to compare CMC and FFS data on multiple measures of completeness (e.g., mean claims per enrollee) and quality (e.g., proportion of claims with a primary diagnosis). We hypothesized that data completeness and quality for CMC enrollees would improve over time, with more states meeting criteria in later years. We also expected that states would have more complete, high‐quality prescription drug data relative to inpatient and outpatient data as pharmacy records track reimbursed, dispensed medications.

## 2 METHODS

### 2.1 Study design and setting

We conducted an observational study of secondary data, analyzing national Medicaid claims from 2001 to 2019. Data were collected longitudinally by states and submitted to CMS. After submission, data were optimized for research on Medicaid enrollment, demographics, health services utilization, and spending across states. Data from 2001 to 2013 were drawn from the MAX research files. During the main transition period in 2014–2015, research files included data in both MAX and TAF formats.^25^ From 2016 to 2019, all data were in TAF format (Table A1). We used the most updated TAF data releases from 2014 to 2019 (Release 2 in 2014–2018, Release 1 in 2019). Data from 2001 to 2013 included 45 states, excluding Arizona, the District of Columbia (DC), Delaware, Nevada, Oregon, and Rhode Island. Data from 2014 to 2019 included all 50 states and DC. TAF files from 2014 to 2019 also capture data from Puerto Rico and the Virgin Islands, which were excluded from these analyses.

### 2.2 Sample selection

The sample included adults aged 18 to 64 years with continuous enrollment in each study year. We applied further restrictions to ensure the sample had an opportunity to contain complete inpatient, outpatient, and prescription drug data for analysis. We limited the sample to individuals who were eligible for the full scope of Medicaid benefits. In addition to excluding older adults (≥65 years) who are eligible for Medicare coverage on the basis of age, we excluded individuals with dual Medicare–Medicaid enrollment for any reason because Medicare is the primary payer for these individuals and their Medicaid data may be incomplete. Although we focused on adults, we also excluded enrollees whose basis of eligibility for Medicaid was classified as child or Children's Health Insurance Program (CHIP). Children were examined in separate analyses^26^ because managed care enrollment rates and data collection, reporting, and quality may differ across eligible groups^14^, ^15^ and health services research often considers developmentally specific diagnosis and treatment measures. Variables used for sample identification are shown in Table A2, with notes on data management procedures to prepare variables for analyses.

### 2.3 Measures and analyses

For the managed care cohorts, analyses were limited to CMC plans because they provide coverage for a complete set of services.^15^ First, we calculated the proportion of enrollees with CMC in each state. For these calculations, the denominator included all individuals meeting study inclusion criteria and the numerator included individuals who were continuously enrolled in a CMC plan for the full calendar year. As in prior studies, states with <10% of enrollees in CMC plans in a given year were excluded from further analyses.^13^, ^14^, ^15^, ^16^

We then examined data for individuals with continuous calendar‐year enrollment in either a CMC or FFS plan, comparing CMC data in states with at least 10% CMC enrollment with FFS data across all available states. Because the goal was to characterize the completeness and quality of CMC data for research purposes, we limited analyses to claims representing health service utilization, excluding records for capitation payments and service tracking. Similar to previous studies, we also restricted analyses to key service types (Table A3).^14^, ^15^, ^16^ For example, analyses of: (1) inpatient files were restricted to stays with at least one claim for inpatient hospital services; (2) outpatient files were restricted to claims for outpatient, physician, advanced practitioner, and clinic visits; and (3) pharmacy files were restricted to claims for prescription drugs (e.g., excluding medical supplies/devices).

We defined two measures of completeness for inpatient, outpatient, and prescription drug data: (1) the proportion of enrollees with any claim and (2) the mean number of claims per enrollee among those with at least one claim. We defined multiple measures of data quality that were also calculated among enrollees with at least one claim. For inpatient and outpatient files, measures included the proportion of claims with: (1) a primary diagnosis code and (2) a procedure code. For pharmacy files, measures included the proportion of claims with: (1) National Drug Codes (NDC), (2) fill dates, (3) days supplied, and (4) quantity dispensed. For the proportion of claims with a primary diagnosis code (inpatient, outpatient), procedure code (inpatient, outpatient), and NDC (pharmacy), we considered a common set of invalid values in addition to missingness as failing to meet criteria, including fields containing all zeros, nines, dots, or spaces. Measure definitions are summarized in Table A4.

Following established methods,^14^, ^15^, ^16^, ^17^ we constructed a range of acceptable values for each measure to determine whether CMC data met completeness and quality criteria. Using FFS data as the reference, we calculated means and standard deviations across all states each year. Similar to a 95% confidence interval, we defined the limits of the acceptable range as the overall FFS mean ± two standard deviations. For each measure, state‐level CMC results were compared with this overall range of acceptable values in each study year to determine data completeness and quality. CMC state‐years within these ranges were considered to have met the criteria for low‐concern data. For each measure, we show the FFS mean and standard deviation used to calculate acceptable ranges and the minimum and maximum values of qualifying low‐concern CMC states (Figures A1–A14).

To summarize trends over time in CMC data completeness and quality at the national level, we calculated the proportion of states meeting the criteria in each year. To estimate the proportion of covered individuals with low‐concern CMC data during the study period, we identified a denominator of adults with at least one month of Medicaid enrollment each year who met inclusion criteria described above for age (18–64 years), basis of eligibility (e.g., excluding child/CHIP), and Medicare enrollment.

Data management and statistical analyses were conducted using SAS Enterprise Guide 8.3.^27^ This study was approved by the Rutgers University Institutional Review Board and followed the REporting of studies Conducted using Observational Routinely‐collected Data (RECORD) guidelines.^28^

## 3 RESULTS

### 3.1 Sample

The sample included 194,364,647 adult Medicaid enrollees who met the inclusion criteria. The share of enrollees in CMC plans increased over the study period, from 36.5% in 2001 to 74.9% in 2019. The final sample for analyses of data completeness and quality included 135,451,659 enrollees after excluding CMC enrollees in states with low (<10%) CMC penetration and restricting to enrollees with continuous calendar‐year enrollment in either a FFS (*n* = 26,600,610; 19.6%) or CMC (*n* = 108,851,049; 80.4%) plan.

### 3.2 Overall completeness and quality of encounter data

**FIGURE 1:**
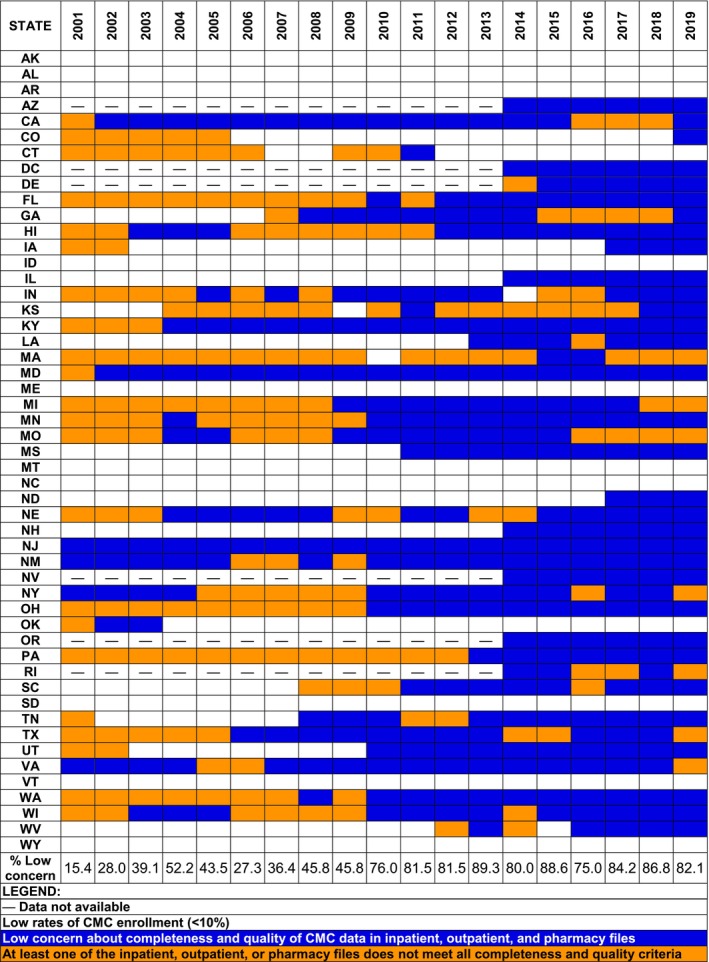
Overall comprehensive managed care (CMC) data completeness and quality compared with fee‐for‐service (FFS) claims. Completeness measures were defined as: (1) the percentage of enrollees with any inpatient, outpatient, or prescription claim and (2) the mean number of inpatient, outpatient, and prescription claims per enrollee with any claim. Quality measures were defined as: (1) the percentage of inpatient/outpatient claims with a primary diagnosis and procedure code and (2) the percentage of prescription claims with a fill date, National Drug Code (NDC), days supplied, and quantity dispensed.

Figure 1 summarizes the findings for CMC data completeness and quality compared with FFS claims for all study measures across inpatient, outpatient, and pharmacy files, showing state‐years with low CMC penetration (<10%) and those with either low‐concern CMC data for all study measures across all files or any concern for any study measure in any file. Of 891 state‐years from 2001 to 2019, over one third (*n* = 351; 39.4%) had low CMC penetration (<10%) and were not further analyzed for data completeness and quality. Of the remaining 540 state‐years with at least 10% CMC enrollment, 349 (64.6%) were identified as low concern for all study measures across all files (Figure 1). Beginning in 2010, over 80% of states met the criteria for low‐concern data completeness and low‐concern data quality across all measures and file types (Figure 1). Figures A1–A14 show the results for individual measures by state and year for each file.

**FIGURE 2:**
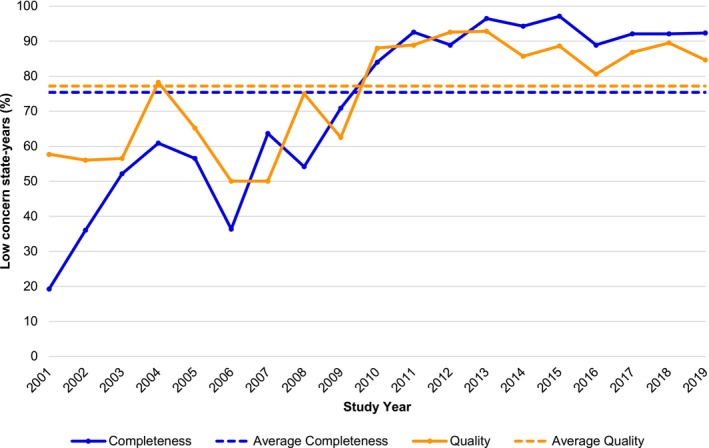
Proportion of analyzed states with low‐concern comprehensive managed care (CMC) data by year, 2001–2019. Analyzed states included only those with ≥10% CMC enrollment. The proportion of all states with either low‐concern CMC data or low CMC enrollment (<10%) is shown in Figure A15. Mean data completeness and quality were calculated as the weighted average across the study period, where weighting reflects the number of state‐years included in the analyses.

Table 1 shows the proportion of state‐years, with at least 10% CMC enrollment that were identified as low concern for study measures in the full study period, and Figure 2 shows the proportion of low‐concern states in each year of the study period. Across all inpatient, outpatient, and pharmacy files from 2001 to 2019, at least 75% of state‐years met the criteria for all measures of CMC data completeness (*n* = 407; 75.4%) and quality (*n* = 417; 77.2%) (Table 1). Overall completeness improved substantially during the study period from 19.2% in 2001 to 92.3% in 2019 (Figure 2). Overall data quality also improved during this period from 57.7% in 2001 to 84.6% in 2019 (Figure 2). However, a peak was observed in 2013, which was the only year with over 90% of state‐years meeting all completeness (96.4%) and all quality (92.9%) criteria. Altogether, low‐concern state‐years comprised 60%, or 283.2 million, of the total number of person‐years analyzed in the 2001–2019 data.

**TABLE 1:** Proportion of analyzed state‐years with low‐concern CMC data, 2001–2019 (*n* = 540).

	Inpatient		Outpatient		Prescription		All Files	
	*N*	%	*N*	%	*N*	%	*N*	%
Overall completeness	430	79.6	467	86.5	459	85.0	407	75.4
Any	484	89.6	470	87.0	475	88.0	—	—
Mean	449	83.1	502	93.0	471	87.2	—	—
Overall quality	449	83.1	511	94.6	528	97.8	417	77.2
Diagnosis	516	95.6	521	96.5	—	—	—	—
Procedure	468	86.7	529	98.0	—	—	—	—
Fill date	—	—	—	—	533	98.7	—	—
NDC	—	—	—	—	530	98.1	—	—
Days supplied	—	—	—	—	531	98.3	—	—
Quantity dispensed	—	—	—	—	532	98.5	—	—

*Note*: Analyzed state‐years included those with ≥10% CMC enrollment. The proportion of all state‐years with either low‐concern CMC data or low CMC enrollment (<10%) is shown in Table A5. Completeness measures were defined as: (1) the percentage of enrollees with any inpatient, outpatient, or prescription drug claim and (2) the mean number of inpatient, outpatient, and prescription drug claims per enrollee with any claim. Quality measures were defined as: (1) the percentage of inpatient/outpatient claims with a primary diagnosis and procedure code and (2) the percentage of prescription drug claims with a fill date, National Drug Code (NDC), days supplied, and quantity dispensed.

Abbreviation: CMC, comprehensive managed care.

Including state‐years with low CMC penetration among those identified as low concern resulted in substantially more state‐years with low‐concern data (Figure 1). Under this definition, over three‐quarters (*n* = 700; 78.6%) of the total 891 state‐years had low‐concern data across all inpatient, outpatient, and prescription drug measures, including 85.1% (*n* = 758) and 86.2% (*n* = 768) for completeness and quality, respectively (Table A5). With increasing CMC penetration during the study period, the proportion of low‐concern data under this definition followed a similar pattern as in state‐years with at least 10% CMC enrollment, increasing over time with only the first study year (2001) having low‐concern data in less than 60% of state‐years (Figure S1). In terms of enrollees, this translates to approximately 74%, or 353.2 million person‐years with low‐concern CMC data in the 2001–2019 research files.

### 3.3 Inpatient files

Compared with FFS data, CMC data in most state‐years were low concern across all measures of inpatient data completeness (*n* = 430; 79.6%) and quality (*n* = 449; 83.1%) (Table 1). For the proportion of enrollees with any inpatient claim, 89.6% (*n* = 484) of state‐years were within the FFS range. For the mean number of claims per enrollee, 83.1% (n = 449) were within the acceptable range. A greater proportion of states had low‐concern CMC data for study measures assessing data quality. Overall, 95.6% (*n* = 516) and 86.7% (*n* = 468) of state‐years were identified as low concern in terms of the proportion of inpatient claims with a primary diagnosis code and procedure code, respectively (Table 1).

Similar to overall CMC data completeness and quality analyses, including state‐years with low CMC penetration among those identified as low concern resulted in a higher proportion of low‐concern data. Using this definition, 781 (87.7%) of the total 891 state‐years were low concern for both completeness measures and 800 (89.8%) were low concern for both measures of data quality (Table A5).

### 3.4 Outpatient files

CMC data completeness and quality were higher in the analyses of outpatient files, with 86.5% (*n* = 467) of state‐years identified as low concern for both completeness measures and 94.6% (*n* = 511) identified as low concern for both measures of data quality (Table 1). For the proportion of enrollees with any outpatient claim, 87.0% (*n* = 470) of state‐years were within the FFS range. For the mean number of claims per enrollee, 93.0% (*n* = 502) were within the acceptable range. Similar to inpatient findings, a greater proportion of states had low‐concern CMC data for study measures assessing outpatient data quality. Overall, 96.5% (*n* = 521) and 98.0% (*n* = 529) of state‐years were identified as low concern in terms of the proportion of claims with a primary diagnosis code and procedure code, respectively (Table 1).

Including state‐years with low CMC penetration among those identified as low concern resulted in 818 (91.8%) of the total 891 state‐years with low‐concern outpatient data for both completeness measures and 862 (96.7%) with low‐concern data for both measures of data quality (Table A5).

### 3.5 Pharmacy files

In the analyses of pharmacy files, 459 (85.0%) state‐years were identified as low concern for both completeness measures (Table 1). Among enrollees with any prescription drug claim, the vast majority of state‐years (*n* = 528; 97.8%) met the criteria for all four measures of data quality (fill date, NDC, days supplied, and quantity dispensed). For the proportion of enrollees with any prescription claim, 88.0% (*n* = 475) of state‐years were within the FFS range. For the mean number of claims per enrollee, 87.2% (*n* = 471) were within the acceptable range. Similar to inpatient and outpatient findings, a greater proportion of states had low‐concern CMC data for study measures assessing prescription data quality. Overall, 98.7% (*n* = 533), 98.1% (*n* = 530), 98.3% (*n* = 531), and 98.5% (*n* = 532) of state‐years were identified as low concern in terms of the proportion of claims with a fill date, NDC, days supplied, and quantity dispensed, respectively (Table 1).

Including state‐years with low CMC penetration among those identified as low concern resulted in 810 (90.9%) of the total 891 state‐years, with low‐concern prescription data for both completeness measures and 879 (98.7%) with low‐concern data across all measures of data quality (Table A5).

## 4 DISCUSSION

We conducted a comprehensive study of CMC data completeness and quality compared with data for Medicaid FFS enrollees in MAX/TAF research files from 2001 to 2019, a period with significant changes in Medicaid programs and administrative data processing. Overall data completeness and quality were relatively high, with marked improvements over time that help mitigate concerns about analyzing CMC data in research involving Medicaid enrollees. These findings are consistent with prior studies,^13^, ^14^, ^15^, ^16^ and build on existing reports in several important ways.

First, we examined an extended time period to characterize long‐term patterns in CMC data completeness and quality. We found that earlier years of the MAX files had more concerning CMC data. However, from 2010 onward, over 80% of state‐years met all inpatient, outpatient, and prescription drug criteria for data completeness and for data quality. Nonetheless, modest declines were observed from 2014 to 2016 that began to rebound in 2017–2019. These declines coincided with state transitions from MSIS to T‐MSIS reporting systems, with 23 states beginning to submit T‐MSIS data in 2014 and 22 states beginning to submit T‐MSIS data in 2015.^25^ Yet, declines were driven by a small number of states; for example, only three states met all completeness and quality criteria in 2013 but not in 2014 (Texas, Wisconsin, West Virginia). Many states had variation over time in meeting all study criteria, suggesting the need for researchers to carefully examine data completeness and quality for individual states, years, and measures of interest. For example, of 18 states with at least 10% CMC enrollment in all study years, only five (Kentucky, Maryland, New Jersey, Ohio, Pennsylvania) had low‐concern CMC data in every year after the first year of meeting all inpatient, outpatient, and prescription drug criteria. There was also substantial variation across states in the total number of years with low‐concern CMC data. Of 18 states with ≥10% CMC in all 19 study years, California, Kentucky, Maryland, New Jersey, New Mexico, and Virginia had at least 15 years with low‐concern CMC data across all inpatient, outpatient, and prescription drug measures. In contrast, Florida, Michigan, Missouri, and Pennsylvania had at least 10 years that did not meet all criteria.

Second, we included data from before, during, and after three key transition periods. Most importantly, findings during the transition to a new state reporting system (MSIS to T‐MSIS) and research file format (MAX to TAF) could facilitate greater understanding of the impact of these large‐scale developments on administrative and research activities. For example, of 28 states analyzed in both their last year of MAX data and first year of TAF data, only five states meeting all CMC data completeness and quality criteria in the last MAX year did not meet all the criteria in the first TAF year (California, Louisiana, Missouri, New York, Wisconsin). Moreover, 22 states had consistent overall findings across the MAX to TAF transition—18 of which had consistently low‐concern CMC data for all criteria—and one state that did not meet all criteria in the last MAX year was low concern in the first TAF year (Massachusetts). Overall, this could indicate that state efforts to adapt to the new system or federal efforts to support state transitions were largely successful, particularly considering there were multiple data releases from 2014 to 2018 to address data quality issues in initial releases.^29^ Another recent study limited to the MAX to TAF transition years also found that the quality of managed care and FFS data were comparable during this period.^30^ More broadly, the study period included years preceding and following the Affordable Care Act Medicaid expansion (2014) and the transition from ICD‐9 to ICD‐10 (2015), which could help researchers identify possible explanations for inconsistent trends or anomalous findings in specific study samples.

Additionally, these findings complement the CMS Data Quality (DQ) Atlas, which provides current information on TAF data quality across a range of topics.^31^ In addition to enrollment benchmarking for key program and plan types, the DQ Atlas assesses the availability of CMC encounter data independent of FFS data. The present findings extend this work by comparing CMC and FFS enrollees to improve the understanding of the completeness and quality of available data within the research files. By restricting the sample to state‐years with ≥10% CMC enrollment, our analyses may reflect states with greater availability of CMC data. However, underreported CMC data are also possible in states with ≥10% CMC penetration. Thus, when designing studies and reporting limitations related to missing or low‐quality data, researchers should consider these findings in conjunction with DQ Atlas information about enrollment and CMC data availability in later TAF years, which could impact the representativeness of study samples and the generalizability of findings to wider Medicaid populations. In research using earlier MAX data, which were not included in the DQ Atlas, researchers should note that state‐years with low‐concern CMC data might nonetheless reflect underreporting and should consider assessing the extent of missing data using other sources. Regardless of the file format or time period, researchers should conduct independent data quality assessments tailored to specific study questions, populations, and measures of interest. Given the complexity of MAX/TAF data, relying solely on comprehensive resources that may not account for key specifications could result in different conclusions about CMC data quality.

For many purposes, national‐level research using available data, particularly in recent years, should be able to retain state‐years representing a large number of enrollees with low‐concern CMC data. Prescription data were particularly low concern, meeting data quality thresholds even in very early MAX years. This is unsurprising as fill dates, drug codes, days supplied, and quantity dispensed are often required for billing purposes.^12^ Indeed, acceptable ranges for these study measures were very narrow, and select state‐years that fell outside the range might be low concern for a particular study question or sample. For example, the FFS mean for prescription drug claims with days supplied was over 99% in all years, with most lower bounds exceeding 95% (Figure A13). Thus, a state with days supplied on 94.9% of CMC claims (i.e., below the acceptable range) might have low‐concern CMC data for a given research question, again highlighting the need to account for study‐specific goals and considerations. Importantly, while we examined key variables often used in research, we did not exhaustively assess the values within data fields. We assessed a set of common invalid entries (e.g., zeros, nines), but diagnosis, procedure, and prescription drug data could still have invalid values (e.g., not corresponding to an existing code or coded using the wrong system). Researchers should conduct detailed data quality checks even among state‐years meeting the criteria in the present analysis to assess the accuracy of reported values for specific health conditions (e.g., diagnoses) or services (e.g., procedures, medications) of interest.^32^, ^33^ Future studies employing logic or consistency checks could further improve the understanding of CMC data and increase confidence in the validity and reliability of these data for research. Specifically, connectivity measures that evaluate the interdependence of clinical diagnoses and services can assess data quality across different file types (e.g., outpatient and prescription claims).^17^ Additionally, external data sources can be used as benchmarks to assess data completeness and quality, as comparable FFS and CMC data may reflect reporting problems across plan types.^30^ For example, comparing state‐level Medicaid data with state‐specific MAX/TAF data could provide some insight on data completeness by plan type (e.g., record volume), and non‐Medicaid administrative datasets (e.g., alternate payers, electronic health records) can be used to evaluate the accuracy of particular diagnosis and service codes.

This study is subject to additional limitations. The sample included 45 states from 2001 to 2013, limiting the understanding of long‐term trends for select states. The sample was restricted to adults and findings may not generalize to child/CHIP enrollees.^26^ We focused on enrollees in CMC plans, which may have higher quality data than other types of managed care.^15^ While future research is needed on other managed care arrangements (e.g., managed behavioral health care), these findings are applicable to a large segment of Medicaid enrollees, as 70% of individuals in this study had continuous calendar‐year enrollment in either a FFS or CMC plan. Relatedly, the sample reflects a population with relatively stable, continuous calendar‐year enrollment. While disenrollment, or “churn,” may be higher in Medicaid than other forms of coverage (i.e., commercial, Medicare), rates within a given year are relatively low. For example, only 8% of adult enrollees under the age of 65 had less than 12 months of enrollment in 2018, with a mean length of continuous coverage >11 months.^34^ Nonetheless, these findings may not generalize to groups with less stable enrollment, which may reflect administrative challenges that could contribute to lower CMC data completeness and quality. Future research is needed to understand data completeness and quality for these groups—particularly in periods when churn might be higher, such as after the COVID‐19 Public Health Emergency ended in 2023 (Medicaid “unwinding”).^35^

Finally, results might differ using different benchmark populations. For example, thresholds for data completeness and quality may underestimate the number of state‐years with low‐concern CMC data because acceptable ranges were calculated using national FFS data. Thus, some states falling outside the national range could have comparable within‐state CMC and FFS data that could potentially be used in research. Alternatively, FFS comparisons may overestimate the number of state‐years with low‐concern CMC data, particularly when FFS variation is high. Because the goal was to understand the extent to which CMC and FFS data were comparable in CMS research files, high variability in study measures among FFS enrollees would allow for similarly high variability among CMC enrollees. This underscores the critical importance of ongoing work to identify and address quality issues in TAF data regardless of plan type. We provide methodological notes for problems identified in select state‐years for variables used in sample identification (Table A2); however, data quality issues in underlying data elements could impact findings and future work could uncover additional issues to consider in determining research design and analytic approach.

## 5 CONCLUSIONS

As one of the main sources of public health insurance coverage in the United States, national Medicaid data are a large, rich source of information for conducting health services and epidemiological research. In this comprehensive study, we found that CMC data in most state‐years were comparable to yearly FFS population data in terms of several measures of data completeness and quality. With detailed information at the state‐year level over a period of tremendous growth in Medicaid managed care, specific results from inpatient, outpatient, and prescription files and measures can help inform the conduct of rigorous research using national MAX/TAF Medicaid data.

## FUNDING INFORMATION

This work was supported by the National Institute on Drug Abuse, grant numbers: K01DA049950 and R01DA047347.

## CONFLICT OF INTEREST STATEMENT

HS has received consulting fees from The Pew Charitable Trusts and the American Society of Addiction Medicine unrelated to this work.

## Supporting information


**Data S1.** Supporting information.
