# Supplementary material for: Completeness and quality of comprehensive managed care data compared with fee‐for‐service data in national Medicaid claims from 2001 to 2019
Source: Health Serv Res. 2025 Jan 2;60(3):e14429. doi: 10.1111/1475-6773.14429 (PMC12120513; doi:10.1111/1475-6773.14429)
Supplement: Supplementary file 1 — Data S1. Supporting information. [file HESR-60-0-s001.docx]

**Appendix**

**Contents:**

[**Table A1. Timeline of Medicaid data reporting and research file formats** 2](#_Toc168992643)

[**Table A2. Variables used for sample identification** 3](#_Toc168992644)

[**Table A3. Type of Service Codes** 4](#_Toc168992645)

[**Table A4. Measure definitions** 4](#_Toc168992646)

[**Figure A1. CMC Results for Percentage of Enrollees with Any Inpatient Claim** 5](#_Toc168992647)

[**Figure A2. CMC Results for Mean Number of Inpatient Claims per Enrollee** 7](#_Toc168992648)

[**Figure A3. CMC Results for Percentage of Inpatient Claims with a Primary Diagnosis** 9](#_Toc168992649)

[**Figure A4. CMC Results for Percentage of Inpatient Claims with a Procedure Code** 11](#_Toc168992650)

[**Figure A5. CMC Results for Percentage of Enrollees with Any Outpatient Claim** 13](#_Toc168992651)

[**Figure A6. CMC Results for Mean Number of Outpatient Claims per Enrollee** 15](#_Toc168992652)

[**Figure A7. CMC Results for Percentage of Outpatient Claims with a Primary Diagnosis** 17](#_Toc168992653)

[**Figure A8. CMC Results for Percentage of Outpatient Claims with a Procedure Code** 19](#_Toc168992654)

[**Figure A9. CMC Results for Percentage of Enrollees with Any Pharmacy Claim** 21](#_Toc168992655)

[**Figure A10. CMC Results for Mean Number of Pharmacy Claims per Enrollee** 23](#_Toc168992656)

[**Figure A11. CMC Results for Percentage of Pharmacy Claims with a Fill Date** 25](#_Toc168992657)

[**Figure A12. CMC Results for Percentage of Pharmacy Claims with a National Drug Code** 27](#_Toc168992658)

[**Figure A13. CMC Results for Percentage of Pharmacy Claims with Days Supplied** 29](#_Toc168992659)

[**Figure A14. CMC Results for Percentage of Pharmacy Claims with Quantity Dispensed** 31](#_Toc168992660)

[**Table A5. Proportion of All State-Years with Low-Concern CMC Data, 2001-2019 (n=891)** 33](#_Toc168992661)

[**Figure A15. Proportion of All States with Low-Concern CMC Data by Year, 2001-2019** 34](#_Toc168992662)

# **Table A1. Timeline of Medicaid data reporting and research file formats**

|  | **2001-2010** | **2011 – 2013** | **2014 – 2015** | **2016 – 2019** |
| --- | --- | --- | --- | --- |
| Reporting system | MSIS | Transition to T-MSIS | | T-MSIS |
| Research file format | MAX | | Transition to TAF | TAF |

# **Table A2. Variables used for sample identification**

|  | **MAX (2001 – 2015)** | **TAF (2014 – 2019)** |
| --- | --- | --- |
| Enrollee identifier | MSIS_ID | MSIS_ID |
| State | STATE_CD | STATE_CD |
| Year | MAX_YR_DT | RFRNC_YR |
| Age | EL_DOB | BIRTH_DT |
| Medicaid enrollment | EL_DAYS_EL_CNT_1 - EL_DAYS_EL_CNT_12 | MDCD_ENRLMT_DAYS_01 - MDCD_ENRLMT_DAYS_12 |
| Medicare eligibility | EL_MDCR_DUAL_MO_1 - EL_MDCR_DUAL_MO_12 | DUAL_ELGBL_CD_01 - DUAL_ELGBL_CD_12 |
| 2001-2005: | EL_MDCR_BEN_MO_1 - EL_MDCR_BEN_MO_12 |  |
| Medicaid basis of eligibility | MAX_ELG_CD_MO_1 - MAX_ELG_CD_MO_12 | ELGBLTY_GRP_CD_01 - ELGBLTY_GRP_CD_12 |
| 2014-2015: | TMSIS_ELG_GRP_MO_1-TMSIS_ELG_GRP_MO_12 |  |
| Scope of benefits | EL_RSTRCT_BNFT_FLG_1 - EL_RSTRCT_BNFT_FLG_12 | RSTRCTD_BNFTS_CD_01 - RSTRCTD_BNFTS_CD_12 |
| Managed care plan | EL_PHP_TYPE_1-4_1 - EL_PHP_TYPE_1-4_12 | MC_PLAN_TYPE_CD_01 - MC_PLAN_TYPE_CD_12 |

**Notes:** In March 2015, Maryland had missing data for several variables in the TAF Personal Summary file used to identify the study population, including Medicaid enrollment, Medicare eligibility, Medicaid basis of eligibility, and scope of benefits. Enrollees who met inclusion criteria in the 11 months with non-missing data were included in the sample. In March and April 2018, Pennsylvania had truncated Medicaid enrollment days in the TAF data, with maximum values of 30 (instead of 31) and 29 (instead of 30), respectively. Enrollees with the maximum value in each month were considered to have continuous enrollment in that month.

# **Table A3. Type of Service Codes**

| **File** | **MAX (2001 – 2015)** | **TAF (2014 – 2019)** |
| --- | --- | --- |
| Inpatient | 01 - Inpatient hospital | 001 - Inpatient hospital services, other than services in an institution for mental diseases  060 - Emergency hospital services  090 - Critical access hospital services - IP |
| Outpatient | 08 – Physicians  11 – Outpatient hospital  12 – Clinic  37 – Nurse practitioner | 002 – Outpatient hospital services  003 – Rural health clinic services  010 – EPSDT services  012 – Physicians’ services  014 – Outpatient substance use treatment services  023 – Advanced practice nurse services  026 – Nurse practitioner services  028 – Clinic services  039 – Diagnostic services  040 – Screening services  041 – Preventive services  042 – Well-baby and well-child care services  049 – Outpatient mental health (excluding outpatient substance use treatment  060 – Emergency hospital services  061 – Critical access hospital services - OT |
| Pharmacy | 16 – Prescribed drugs | 33 – Prescribed drugs |

# **Table A4. Measure definitions**

| **Completeness** | **Medicaid files** |
| --- | --- |
| % enrollees with any claims | Inpatient, outpatient, pharmacy |
| Mean claims per enrollee | Inpatient, outpatient, pharmacy |
| **Quality** |  |
| % claims with primary diagnosis code | Inpatient, outpatient |
| % claims with primary procedure code | Inpatient, outpatient |
| % claims with date prescribed | Pharmacy |
| % claims with NDC number | Pharmacy |
| % claims with days supplied | Pharmacy |
| % claims with quantity | Pharmacy |

# **Figure A1. CMC Results for Percentage of Enrollees with Any Inpatient Claim**

| **STATE** | | **2001** | **2002** | **2003** | **2004** | **2005** | **2006** | **2007** | **2008** | **2009** | **2010** | **2011** | **2012** | **2013** | **2014** | **2015** | **2016** | **2017** | **2018** | **2019** |
| --- | --- | --- | --- | --- | --- | --- | --- | --- | --- | --- | --- | --- | --- | --- | --- | --- | --- | --- | --- | --- |
| **AK** | |  |  |  |  |  |  |  |  |  |  |  |  |  |  |  |  |  |  |  |
| **AL** | |  |  |  |  |  |  |  |  |  |  |  |  |  |  |  |  |  |  |  |
| **AR** | |  |  |  |  |  |  |  |  |  |  |  |  |  |  |  |  |  |  |  |
| **AZ** | | — | — | — | — | — | — | — | — | — | — | — | — | — |  |  |  |  |  |  |
| **CA** | |  |  |  |  |  |  |  |  |  |  |  |  |  |  |  |  |  |  |  |
| **CO** | |  |  |  |  |  |  |  |  |  |  |  |  |  |  |  |  |  |  |  |
| **CT** | |  |  |  |  |  |  |  |  |  |  |  |  |  |  |  |  |  |  |  |
| **DC** | | — | — | — | — | — | — | — | — | — | — | — | — | — |  |  |  |  |  |  |
| **DE** | | — | — | — | — | — | — | — | — | — | — | — | — | — |  |  |  |  |  |  |
| **FL** | |  |  |  |  |  |  |  |  |  |  |  |  |  |  |  |  |  |  |  |
| **GA** | |  |  |  |  |  |  |  |  |  |  |  |  |  |  |  |  |  |  |  |
| **HI** | |  |  |  |  |  |  |  |  |  |  |  |  |  |  |  |  |  |  |  |
| **IA** | |  |  |  |  |  |  |  |  |  |  |  |  |  |  |  |  |  |  |  |
| **ID** | |  |  |  |  |  |  |  |  |  |  |  |  |  |  |  |  |  |  |  |
| **IL** | |  |  |  |  |  |  |  |  |  |  |  |  |  |  |  |  |  |  |  |
| **IN** | |  |  |  |  |  |  |  |  |  |  |  |  |  |  |  |  |  |  |  |
| **KS** | |  |  |  |  |  |  |  |  |  |  |  |  |  |  |  |  |  |  |  |
| **KY** | |  |  |  |  |  |  |  |  |  |  |  |  |  |  |  |  |  |  |  |
| **LA** | |  |  |  |  |  |  |  |  |  |  |  |  |  |  |  |  |  |  |  |
| **MA** | |  |  |  |  |  |  |  |  |  |  |  |  |  |  |  |  |  |  |  |
| **MD** | |  |  |  |  |  |  |  |  |  |  |  |  |  |  |  |  |  |  |  |
| **ME** | |  |  |  |  |  |  |  |  |  |  |  |  |  |  |  |  |  |  |  |
| **MI** | |  |  |  |  |  |  |  |  |  |  |  |  |  |  |  |  |  |  |  |
| **MN** | |  |  |  |  |  |  |  |  |  |  |  |  |  |  |  |  |  |  |  |
| **MO** | |  |  |  |  |  |  |  |  |  |  |  |  |  |  |  |  |  |  |  |
| **MS** | |  |  |  |  |  |  |  |  |  |  |  |  |  |  |  |  |  |  |  |
| **MT** | |  |  |  |  |  |  |  |  |  |  |  |  |  |  |  |  |  |  |  |
| **NC** | |  |  |  |  |  |  |  |  |  |  |  |  |  |  |  |  |  |  |  |
| **ND** | |  |  |  |  |  |  |  |  |  |  |  |  |  |  |  |  |  |  |  |
| **NE** | |  |  |  |  |  |  |  |  |  |  |  |  |  |  |  |  |  |  |  |
| **NH** | |  |  |  |  |  |  |  |  |  |  |  |  |  |  |  |  |  |  |  |
| **NJ** | |  |  |  |  |  |  |  |  |  |  |  |  |  |  |  |  |  |  |  |
| **NM** | |  |  |  |  |  |  |  |  |  |  |  |  |  |  |  |  |  |  |  |
| **NV** | | — | — | — | — | — | — | — | — | — | — | — | — | — |  |  |  |  |  |  |
| **NY** | |  |  |  |  |  |  |  |  |  |  |  |  |  |  |  |  |  |  |  |
| **OH** | |  |  |  |  |  |  |  |  |  |  |  |  |  |  |  |  |  |  |  |
| **OK** | |  |  |  |  |  |  |  |  |  |  |  |  |  |  |  |  |  |  |  |
| **OR** | | — | — | — | — | — | — | — | — | — | — | — | — | — |  |  |  |  |  |  |
| **PA** | |  |  |  |  |  |  |  |  |  |  |  |  |  |  |  |  |  |  |  |
| **RI** | | — | — | — | — | — | — | — | — | — | — | — | — | — |  |  |  |  |  |  |
| **SC** | |  |  |  |  |  |  |  |  |  |  |  |  |  |  |  |  |  |  |  |
| **SD** | |  |  |  |  |  |  |  |  |  |  |  |  |  |  |  |  |  |  |  |
| **TN** | |  |  |  |  |  |  |  |  |  |  |  |  |  |  |  |  |  |  |  |
| **TX** | |  |  |  |  |  |  |  |  |  |  |  |  |  |  |  |  |  |  |  |
| **UT** | |  |  |  |  |  |  |  |  |  |  |  |  |  |  |  |  |  |  |  |
| **VA** | |  |  |  |  |  |  |  |  |  |  |  |  |  |  |  |  |  |  |  |
| **VT** | |  |  |  |  |  |  |  |  |  |  |  |  |  |  |  |  |  |  |  |
| **WA** | |  |  |  |  |  |  |  |  |  |  |  |  |  |  |  |  |  |  |  |
| **WI** | |  |  |  |  |  |  |  |  |  |  |  |  |  |  |  |  |  |  |  |
| **WV** | |  |  |  |  |  |  |  |  |  |  |  |  |  |  |  |  |  |  |  |
| **WY** | |  |  |  |  |  |  |  |  |  |  |  |  |  |  |  |  |  |  |  |
| **FFS** | **Mean** | 19.0 | 19.6 | 17.6 | 17.0 | 16.2 | 15.9 | 16.0 | 14.0 | 14.2 | 13.2 | 13.9 | 13.0 | 12.0 | 12.0 | 9.7 | 8.2 | 9.1 | 8.7 | 8.3 |
|  | **SD** | 5.4 | 9.6 | 9.0 | 5.6 | 6.8 | 7.7 | 7.2 | 7.7 | 7.0 | 8.3 | 8.0 | 7.2 | 7.3 | 7.1 | 5.5 | 5.0 | 7.2 | 8.8 | 6.7 |
| **CMC** | **Min** | 11.7 | 0.6 | <0.1 | 6.8 | 6.8 | 0.9 | 2.2 | <0.1 | 1.7 | 0.0 | <0.1 | 1.0 | 0.9 | 1.0 | 0.1 | 0.1 | 4.2 | 0.5 | 2.4 |
|  | **Max** | 15.3 | 16.8 | 19.1 | 22.4 | 23.2 | 22.3 | 23.5 | 23.5 | 22.8 | 22.9 | 19.0 | 19.3 | 19.6 | 17.1 | 18.1 | 17.7 | 19.8 | 19.4 | 19.0 |
| **LEGEND:** | | | | | | | | | | | | | | | | | | | | |
| **—** **Data not available** | | | | | | | | | | | | | | | | | | | | |
| **Low rates (<10%) of CMC enrollment** | | | | | | | | | | | | | | | | | | | | |
| **Low concern** | | | | | | | | | | | | | | | | | | | | |
| **Did not meet criteria** | | | | | | | | | | | | | | | | | | | | |

**Abbreviations:** CMC = comprehensive managed care; FFS = fee-for-service; SD = standard deviation

**Notes:** Measure was defined as the percentage of enrollees with any inpatient claim. Inpatient files were analyzed at the stay level for consistency, as MAX records were reported only at the stay level. State-years were identified as low concern if data for CMC enrollees were comparable to (i.e. within 2 standard deviations of) the national FFS population in the year.

# **Figure A2. CMC Results for Mean Number of Inpatient Claims per Enrollee**

| **STATE** | | **2001** | **2002** | **2003** | **2004** | **2005** | **2006** | **2007** | **2008** | **2009** | **2010** | **2011** | **2012** | **2013** | **2014** | **2015** | **2016** | **2017** | **2018** | **2019** |
| --- | --- | --- | --- | --- | --- | --- | --- | --- | --- | --- | --- | --- | --- | --- | --- | --- | --- | --- | --- | --- |
| **AK** | |  |  |  |  |  |  |  |  |  |  |  |  |  |  |  |  |  |  |  |
| **AL** | |  |  |  |  |  |  |  |  |  |  |  |  |  |  |  |  |  |  |  |
| **AR** | |  |  |  |  |  |  |  |  |  |  |  |  |  |  |  |  |  |  |  |
| **AZ** | | — | — | — | — | — | — | — | — | — | — | — | — | — |  |  |  |  |  |  |
| **CA** | |  |  |  |  |  |  |  |  |  |  |  |  |  |  |  |  |  |  |  |
| **CO** | |  |  |  |  |  |  |  |  |  |  |  |  |  |  |  |  |  |  |  |
| **CT** | |  |  |  |  |  |  |  |  |  |  |  |  |  |  |  |  |  |  |  |
| **DC** | | — | — | — | — | — | — | — | — | — | — | — | — | — |  |  |  |  |  |  |
| **DE** | | — | — | — | — | — | — | — | — | — | — | — | — | — |  |  |  |  |  |  |
| **FL** | |  |  |  |  |  |  |  |  |  |  |  |  |  |  |  |  |  |  |  |
| **GA** | |  |  |  |  |  |  |  |  |  |  |  |  |  |  |  |  |  |  |  |
| **HI** | |  |  |  |  |  |  |  |  |  |  |  |  |  |  |  |  |  |  |  |
| **IA** | |  |  |  |  |  |  |  |  |  |  |  |  |  |  |  |  |  |  |  |
| **ID** | |  |  |  |  |  |  |  |  |  |  |  |  |  |  |  |  |  |  |  |
| **IL** | |  |  |  |  |  |  |  |  |  |  |  |  |  |  |  |  |  |  |  |
| **IN** | |  |  |  |  |  |  |  |  |  |  |  |  |  |  |  |  |  |  |  |
| **KS** | |  |  |  |  |  |  |  |  |  |  |  |  |  |  |  |  |  |  |  |
| **KY** | |  |  |  |  |  |  |  |  |  |  |  |  |  |  |  |  |  |  |  |
| **LA** | |  |  |  |  |  |  |  |  |  |  |  |  |  |  |  |  |  |  |  |
| **MA** | |  |  |  |  |  |  |  |  |  |  |  |  |  |  |  |  |  |  |  |
| **MD** | |  |  |  |  |  |  |  |  |  |  |  |  |  |  |  |  |  |  |  |
| **ME** | |  |  |  |  |  |  |  |  |  |  |  |  |  |  |  |  |  |  |  |
| **MI** | |  |  |  |  |  |  |  |  |  |  |  |  |  |  |  |  |  |  |  |
| **MN** | |  |  |  |  |  |  |  |  |  |  |  |  |  |  |  |  |  |  |  |
| **MO** | |  |  |  |  |  |  |  |  |  |  |  |  |  |  |  |  |  |  |  |
| **MS** | |  |  |  |  |  |  |  |  |  |  |  |  |  |  |  |  |  |  |  |
| **MT** | |  |  |  |  |  |  |  |  |  |  |  |  |  |  |  |  |  |  |  |
| **NC** | |  |  |  |  |  |  |  |  |  |  |  |  |  |  |  |  |  |  |  |
| **ND** | |  |  |  |  |  |  |  |  |  |  |  |  |  |  |  |  |  |  |  |
| **NE** | |  |  |  |  |  |  |  |  |  |  |  |  |  |  |  |  |  |  |  |
| **NH** | |  |  |  |  |  |  |  |  |  |  |  |  |  |  |  |  |  |  |  |
| **NJ** | |  |  |  |  |  |  |  |  |  |  |  |  |  |  |  |  |  |  |  |
| **NM** | |  |  |  |  |  |  |  |  |  |  |  |  |  |  |  |  |  |  |  |
| **NV** | | — | — | — | — | — | — | — | — | — | — | — | — | — |  |  |  |  |  |  |
| **NY** | |  |  |  |  |  |  |  |  |  |  |  |  |  |  |  |  |  |  |  |
| **OH** | |  |  |  |  |  |  |  |  |  |  |  |  |  |  |  |  |  |  |  |
| **OK** | |  |  |  |  |  |  |  |  |  |  |  |  |  |  |  |  |  |  |  |
| **OR** | | — | — | — | — | — | — | — | — | — | — | — | — | — |  |  |  |  |  |  |
| **PA** | |  |  |  |  |  |  |  |  |  |  |  |  |  |  |  |  |  |  |  |
| **RI** | | — | — | — | — | — | — | — | — | — | — | — | — | — |  |  |  |  |  |  |
| **SC** | |  |  |  |  |  |  |  |  |  |  |  |  |  |  |  |  |  |  |  |
| **SD** | |  |  |  |  |  |  |  |  |  |  |  |  |  |  |  |  |  |  |  |
| **TN** | |  |  |  |  |  |  |  |  |  |  |  |  |  |  |  |  |  |  |  |
| **TX** | |  |  |  |  |  |  |  |  |  |  |  |  |  |  |  |  |  |  |  |
| **UT** | |  |  |  |  |  |  |  |  |  |  |  |  |  |  |  |  |  |  |  |
| **VA** | |  |  |  |  |  |  |  |  |  |  |  |  |  |  |  |  |  |  |  |
| **VT** | |  |  |  |  |  |  |  |  |  |  |  |  |  |  |  |  |  |  |  |
| **WA** | |  |  |  |  |  |  |  |  |  |  |  |  |  |  |  |  |  |  |  |
| **WI** | |  |  |  |  |  |  |  |  |  |  |  |  |  |  |  |  |  |  |  |
| **WV** | |  |  |  |  |  |  |  |  |  |  |  |  |  |  |  |  |  |  |  |
| **WY** | |  |  |  |  |  |  |  |  |  |  |  |  |  |  |  |  |  |  |  |
| **FFS** | **Mean** | 1.8 | 1.8 | 1.7 | 1.7 | 1.8 | 1.8 | 1.8 | 1.9 | 1.8 | 1.8 | 1.8 | 1.7 | 2.0 | 1.9 | 1.7 | 1.7 | 1.8 | 1.9 | 1.7 |
|  | **SD** | 0.2 | 0.3 | 0.3 | 0.3 | 0.3 | 0.3 | 0.4 | 0.3 | 0.3 | 0.4 | 0.4 | 0.4 | 1.0 | 1.2 | 0.4 | 0.4 | 0.8 | 1.2 | 0.4 |
| **CMC** | **Min** | 1.3 | 1.4 | 1.2 | 1.1 | 1.2 | 1.4 | 1.2 | 1.2 | 1.1 | 1.0 | 1.1 | 1.2 | 1.1 | 1.1 | 1.2 | 1.2 | 1.2 | 1.1 | 1.2 |
|  | **Max** | 1.7 | 1.8 | 1.9 | 1.8 | 2.1 | 2.0 | 2.4 | 1.9 | 1.9 | 2.5 | 2.2 | 2.1 | 2.2 | 2.2 | 2.3 | 2.1 | 2.8 | 4.0 | 2.0 |
| **LEGEND:** | | | | | | | | | | | | | | | | | | | | |
| **— Data not available** | | | | | | | | | | | | | | | | | | | | |
| **Low rates (<10%) of CMC enrollment** | | | | | | | | | | | | | | | | | | | | |
| **Low concern** | | | | | | | | | | | | | | | | | | | | |
| **Did not meet criteria** | | | | | | | | | | | | | | | | | | | | |

**Abbreviations:** CMC = comprehensive managed care; FFS = fee-for-service; SD = standard deviation

**Notes:** Measure was defined as the mean number of inpatient claims per enrollee with >1 inpatient claim. Inpatient files were analyzed at the stay level for consistency, as MAX records were reported only at the stay level. State-years were identified as low concern if data for CMC enrollees were comparable to (i.e. within 2 standard deviations of) the national FFS population in the year.

# **Figure A3. CMC Results for Percentage of Inpatient Claims with a Primary Diagnosis**

| **STATE** | | **2001** | **2002** | **2003** | **2004** | **2005** | **2006** | **2007** | **2008** | **2009** | **2010** | **2011** | **2012** | **2013** | **2014** | **2015** | **2016** | **2017** | **2018** | **2019** |
| --- | --- | --- | --- | --- | --- | --- | --- | --- | --- | --- | --- | --- | --- | --- | --- | --- | --- | --- | --- | --- |
| **AK** | |  |  |  |  |  |  |  |  |  |  |  |  |  |  |  |  |  |  |  |
| **AL** | |  |  |  |  |  |  |  |  |  |  |  |  |  |  |  |  |  |  |  |
| **AR** | |  |  |  |  |  |  |  |  |  |  |  |  |  |  |  |  |  |  |  |
| **AZ** | | — | — | — | — | — | — | — | — | — | — | — | — | — |  |  |  |  |  |  |
| **CA** | |  |  |  |  |  |  |  |  |  |  |  |  |  |  |  |  |  |  |  |
| **CO** | |  |  |  |  |  |  |  |  |  |  |  |  |  |  |  |  |  |  |  |
| **CT** | |  |  |  |  |  |  |  |  |  |  |  |  |  |  |  |  |  |  |  |
| **DC** | | — | — | — | — | — | — | — | — | — | — | — | — | — |  |  |  |  |  |  |
| **DE** | | — | — | — | — | — | — | — | — | — | — | — | — | — |  |  |  |  |  |  |
| **FL** | |  |  |  |  |  |  |  |  |  |  |  |  |  |  |  |  |  |  |  |
| **GA** | |  |  |  |  |  |  |  |  |  |  |  |  |  |  |  |  |  |  |  |
| **HI** | |  |  |  |  |  |  |  |  |  |  |  |  |  |  |  |  |  |  |  |
| **IA** | |  |  |  |  |  |  |  |  |  |  |  |  |  |  |  |  |  |  |  |
| **ID** | |  |  |  |  |  |  |  |  |  |  |  |  |  |  |  |  |  |  |  |
| **IL** | |  |  |  |  |  |  |  |  |  |  |  |  |  |  |  |  |  |  |  |
| **IN** | |  |  |  |  |  |  |  |  |  |  |  |  |  |  |  |  |  |  |  |
| **KS** | |  |  |  |  |  |  |  |  |  |  |  |  |  |  |  |  |  |  |  |
| **KY** | |  |  |  |  |  |  |  |  |  |  |  |  |  |  |  |  |  |  |  |
| **LA** | |  |  |  |  |  |  |  |  |  |  |  |  |  |  |  |  |  |  |  |
| **MA** | |  |  |  |  |  |  |  |  |  |  |  |  |  |  |  |  |  |  |  |
| **MD** | |  |  |  |  |  |  |  |  |  |  |  |  |  |  |  |  |  |  |  |
| **ME** | |  |  |  |  |  |  |  |  |  |  |  |  |  |  |  |  |  |  |  |
| **MI** | |  |  |  |  |  |  |  |  |  |  |  |  |  |  |  |  |  |  |  |
| **MN** | |  |  |  |  |  |  |  |  |  |  |  |  |  |  |  |  |  |  |  |
| **MO** | |  |  |  |  |  |  |  |  |  |  |  |  |  |  |  |  |  |  |  |
| **MS** | |  |  |  |  |  |  |  |  |  |  |  |  |  |  |  |  |  |  |  |
| **MT** | |  |  |  |  |  |  |  |  |  |  |  |  |  |  |  |  |  |  |  |
| **NC** | |  |  |  |  |  |  |  |  |  |  |  |  |  |  |  |  |  |  |  |
| **ND** | |  |  |  |  |  |  |  |  |  |  |  |  |  |  |  |  |  |  |  |
| **NE** | |  |  |  |  |  |  |  |  |  |  |  |  |  |  |  |  |  |  |  |
| **NH** | |  |  |  |  |  |  |  |  |  |  |  |  |  |  |  |  |  |  |  |
| **NJ** | |  |  |  |  |  |  |  |  |  |  |  |  |  |  |  |  |  |  |  |
| **NM** | |  |  |  |  |  |  |  |  |  |  |  |  |  |  |  |  |  |  |  |
| **NV** | | — | — | — | — | — | — | — | — | — | — | — | — | — |  |  |  |  |  |  |
| **NY** | |  |  |  |  |  |  |  |  |  |  |  |  |  |  |  |  |  |  |  |
| **OH** | |  |  |  |  |  |  |  |  |  |  |  |  |  |  |  |  |  |  |  |
| **OK** | |  |  |  |  |  |  |  |  |  |  |  |  |  |  |  |  |  |  |  |
| **OR** | | — | — | — | — | — | — | — | — | — | — | — | — | — |  |  |  |  |  |  |
| **PA** | |  |  |  |  |  |  |  |  |  |  |  |  |  |  |  |  |  |  |  |
| **RI** | | — | — | — | — | — | — | — | — | — | — | — | — | — |  |  |  |  |  |  |
| **SC** | |  |  |  |  |  |  |  |  |  |  |  |  |  |  |  |  |  |  |  |
| **SD** | |  |  |  |  |  |  |  |  |  |  |  |  |  |  |  |  |  |  |  |
| **TN** | |  |  |  |  |  |  |  |  |  |  |  |  |  |  |  |  |  |  |  |
| **TX** | |  |  |  |  |  |  |  |  |  |  |  |  |  |  |  |  |  |  |  |
| **UT** | |  |  |  |  |  |  |  |  |  |  |  |  |  |  |  |  |  |  |  |
| **VA** | |  |  |  |  |  |  |  |  |  |  |  |  |  |  |  |  |  |  |  |
| **VT** | |  |  |  |  |  |  |  |  |  |  |  |  |  |  |  |  |  |  |  |
| **WA** | |  |  |  |  |  |  |  |  |  |  |  |  |  |  |  |  |  |  |  |
| **WI** | |  |  |  |  |  |  |  |  |  |  |  |  |  |  |  |  |  |  |  |
| **WV** | |  |  |  |  |  |  |  |  |  |  |  |  |  |  |  |  |  |  |  |
| **WY** | |  |  |  |  |  |  |  |  |  |  |  |  |  |  |  |  |  |  |  |
| **FFS** | **Mean** | 99.5 | 99.9 | 99.7 | 99.8 | 100 | 100 | 100 | 100 | 100 | 100 | 100 | 99.9 | 100 | 99.5 | 99.8 | 99.9 | 99.9 | 99.9 | 100 |
|  | **SD** | 1.8 | 0.3 | 0.8 | 0.9 | 0.1 | 0.0 | 0.0 | 0.1 | 0.0 | 0.0 | 0.0 | 0.3 | 0.2 | 3.4 | 1.4 | 0.3 | 0.7 | 0.5 | 0.1 |
| **CMC** | **Min** | 99.9 | 99.8 | 99.9 | 100 | 100 | 100 | 100 | 100 | 100 | 100 | 100 | 100 | 99.6 | 95.2 | 98.6 | 99.7 | 100 | 100 | 100 |
|  | **Max** | 100 | 100 | 100 | 100 | 100 | 100 | 100 | 100 | 100 | 100 | 100 | 100 | 100 | 100 | 100 | 100 | 100 | 100 | 100 |
| **LEGEND:** | | | | | | | | | | | | | | | | | | | | |
| **—** **Data not available** | | | | | | | | | | | | | | | | | | | | |
| **Low rates (<10%) of CMC enrollment** | | | | | | | | | | | | | | | | | | | | |
| **Low concern** | | | | | | | | | | | | | | | | | | | | |
| **Did not meet criteria** | | | | | | | | | | | | | | | | | | | | |

**Abbreviations:** CMC = comprehensive managed care; FFS = fee-for-service; SD = standard deviation

**Notes:** Measure was defined as the percentage of inpatient claims with a non-missing value in the first diagnosis code field, considering fields with all zeros, nines, dots, or spaces as missing. Inpatient files were analyzed at the stay level for consistency, as MAX records were reported only at the stay level. State-years were identified as low concern if data for CMC enrollees were comparable to (i.e. within 2 standard deviations of) the national FFS population in the year.

# **Figure A4. CMC Results for Percentage of Inpatient Claims with a Procedure Code**

| **STATE** | | **2001** | **2002** | **2003** | **2004** | **2005** | **2006** | **2007** | **2008** | **2009** | **2010** | **2011** | **2012** | **2013** | **2014** | **2015** | **2016** | **2017** | **2018** | **2019** |
| --- | --- | --- | --- | --- | --- | --- | --- | --- | --- | --- | --- | --- | --- | --- | --- | --- | --- | --- | --- | --- |
| **AK** | |  |  |  |  |  |  |  |  |  |  |  |  |  |  |  |  |  |  |  |
| **AL** | |  |  |  |  |  |  |  |  |  |  |  |  |  |  |  |  |  |  |  |
| **AR** | |  |  |  |  |  |  |  |  |  |  |  |  |  |  |  |  |  |  |  |
| **AZ** | | — | — | — | — | — | — | — | — | — | — | — | — | — |  |  |  |  |  |  |
| **CA** | |  |  |  |  |  |  |  |  |  |  |  |  |  |  |  |  |  |  |  |
| **CO** | |  |  |  |  |  |  |  |  |  |  |  |  |  |  |  |  |  |  |  |
| **CT** | |  |  |  |  |  |  |  |  |  |  |  |  |  |  |  |  |  |  |  |
| **DC** | | — | — | — | — | — | — | — | — | — | — | — | — | — |  |  |  |  |  |  |
| **DE** | | — | — | — | — | — | — | — | — | — | — | — | — | — |  |  |  |  |  |  |
| **FL** | |  |  |  |  |  |  |  |  |  |  |  |  |  |  |  |  |  |  |  |
| **GA** | |  |  |  |  |  |  |  |  |  |  |  |  |  |  |  |  |  |  |  |
| **HI** | |  |  |  |  |  |  |  |  |  |  |  |  |  |  |  |  |  |  |  |
| **IA** | |  |  |  |  |  |  |  |  |  |  |  |  |  |  |  |  |  |  |  |
| **ID** | |  |  |  |  |  |  |  |  |  |  |  |  |  |  |  |  |  |  |  |
| **IL** | |  |  |  |  |  |  |  |  |  |  |  |  |  |  |  |  |  |  |  |
| **IN** | |  |  |  |  |  |  |  |  |  |  |  |  |  |  |  |  |  |  |  |
| **KS** | |  |  |  |  |  |  |  |  |  |  |  |  |  |  |  |  |  |  |  |
| **KY** | |  |  |  |  |  |  |  |  |  |  |  |  |  |  |  |  |  |  |  |
| **LA** | |  |  |  |  |  |  |  |  |  |  |  |  |  |  |  |  |  |  |  |
| **MA** | |  |  |  |  |  |  |  |  |  |  |  |  |  |  |  |  |  |  |  |
| **MD** | |  |  |  |  |  |  |  |  |  |  |  |  |  |  |  |  |  |  |  |
| **ME** | |  |  |  |  |  |  |  |  |  |  |  |  |  |  |  |  |  |  |  |
| **MI** | |  |  |  |  |  |  |  |  |  |  |  |  |  |  |  |  |  |  |  |
| **MN** | |  |  |  |  |  |  |  |  |  |  |  |  |  |  |  |  |  |  |  |
| **MO** | |  |  |  |  |  |  |  |  |  |  |  |  |  |  |  |  |  |  |  |
| **MS** | |  |  |  |  |  |  |  |  |  |  |  |  |  |  |  |  |  |  |  |
| **MT** | |  |  |  |  |  |  |  |  |  |  |  |  |  |  |  |  |  |  |  |
| **NC** | |  |  |  |  |  |  |  |  |  |  |  |  |  |  |  |  |  |  |  |
| **ND** | |  |  |  |  |  |  |  |  |  |  |  |  |  |  |  |  |  |  |  |
| **NE** | |  |  |  |  |  |  |  |  |  |  |  |  |  |  |  |  |  |  |  |
| **NH** | |  |  |  |  |  |  |  |  |  |  |  |  |  |  |  |  |  |  |  |
| **NJ** | |  |  |  |  |  |  |  |  |  |  |  |  |  |  |  |  |  |  |  |
| **NM** | |  |  |  |  |  |  |  |  |  |  |  |  |  |  |  |  |  |  |  |
| **NV** | | — | — | — | — | — | — | — | — | — | — | — | — | — |  |  |  |  |  |  |
| **NY** | |  |  |  |  |  |  |  |  |  |  |  |  |  |  |  |  |  |  |  |
| **OH** | |  |  |  |  |  |  |  |  |  |  |  |  |  |  |  |  |  |  |  |
| **OK** | |  |  |  |  |  |  |  |  |  |  |  |  |  |  |  |  |  |  |  |
| **OR** | | — | — | — | — | — | — | — | — | — | — | — | — | — |  |  |  |  |  |  |
| **PA** | |  |  |  |  |  |  |  |  |  |  |  |  |  |  |  |  |  |  |  |
| **RI** | | — | — | — | — | — | — | — | — | — | — | — | — | — |  |  |  |  |  |  |
| **SC** | |  |  |  |  |  |  |  |  |  |  |  |  |  |  |  |  |  |  |  |
| **SD** | |  |  |  |  |  |  |  |  |  |  |  |  |  |  |  |  |  |  |  |
| **TN** | |  |  |  |  |  |  |  |  |  |  |  |  |  |  |  |  |  |  |  |
| **TX** | |  |  |  |  |  |  |  |  |  |  |  |  |  |  |  |  |  |  |  |
| **UT** | |  |  |  |  |  |  |  |  |  |  |  |  |  |  |  |  |  |  |  |
| **VA** | |  |  |  |  |  |  |  |  |  |  |  |  |  |  |  |  |  |  |  |
| **VT** | |  |  |  |  |  |  |  |  |  |  |  |  |  |  |  |  |  |  |  |
| **WA** | |  |  |  |  |  |  |  |  |  |  |  |  |  |  |  |  |  |  |  |
| **WI** | |  |  |  |  |  |  |  |  |  |  |  |  |  |  |  |  |  |  |  |
| **WV** | |  |  |  |  |  |  |  |  |  |  |  |  |  |  |  |  |  |  |  |
| **WY** | |  |  |  |  |  |  |  |  |  |  |  |  |  |  |  |  |  |  |  |
| **FFS** | **Mean** | 49.1 | 50.8 | 50.9 | 48.0 | 51.1 | 51.1 | 48.8 | 50.7 | 51.1 | 52.7 | 48.6 | 53.4 | 51.2 | 50.0 | 50.8 | 51.7 | 51.3 | 50.0 | 51.3 |
|  | **SD** | 10.2 | 13.5 | 12.2 | 14.4 | 12.7 | 8.7 | 12.4 | 13.1 | 9.6 | 15.1 | 17.9 | 16.3 | 14.3 | 18.8 | 14.2 | 14.8 | 13.2 | 15.7 | 13.6 |
| **CMC** | **Min** | 31.6 | 30.6 | 31.9 | 19.2 | 35.8 | 42.1 | 26.7 | 26.4 | 37.2 | 25.8 | 21.4 | 22.4 | 26.4 | 26.4 | 26.7 | 33.0 | 32.0 | 31.3 | 32.0 |
|  | **Max** | 68.0 | 68.9 | 71.6 | 72.8 | 73.1 | 68.4 | 71.8 | 76.4 | 69.8 | 77.9 | 77.8 | 74.4 | 75.5 | 75.7 | 69.2 | 69.4 | 68.4 | 76.8 | 76.2 |
| **LEGEND:** | | | | | | | | | | | | | | | | | | | | |
| **—** **Data not available** | | | | | | | | | | | | | | | | | | | | |
| **Low rates (<10%) of CMC enrollment** | | | | | | | | | | | | | | | | | | | | |
| **Low concern** | | | | | | | | | | | | | | | | | | | | |
| **Did not meet criteria** | | | | | | | | | | | | | | | | | | | | |

**Abbreviations:** CMC = comprehensive managed care; FFS = fee-for-service; SD = standard deviation

**Notes:** Measure was defined as the percentage of inpatient claims with a non-missing value in the first procedure code field, considering fields with all zeros, nines, dots, or spaces as missing. Inpatient files were analyzed at the stay level for consistency, as MAX records were reported only at the stay level. State-years were identified as low concern if data for CMC enrollees were comparable to (i.e. within 2 standard deviations of) the national FFS population in the year.

# **Figure A5. CMC Results for Percentage of Enrollees with Any Outpatient Claim**

| **STATE** | | **2001** | **2002** | **2003** | **2004** | **2005** | **2006** | **2007** | **2008** | **2009** | **2010** | **2011** | **2012** | **2013** | **2014** | **2015** | **2016** | **2017** | **2018** | **2019** |
| --- | --- | --- | --- | --- | --- | --- | --- | --- | --- | --- | --- | --- | --- | --- | --- | --- | --- | --- | --- | --- |
| **AK** | |  |  |  |  |  |  |  |  |  |  |  |  |  |  |  |  |  |  |  |
| **AL** | |  |  |  |  |  |  |  |  |  |  |  |  |  |  |  |  |  |  |  |
| **AR** | |  |  |  |  |  |  |  |  |  |  |  |  |  |  |  |  |  |  |  |
| **AZ** | | — | — | — | — | — | — | — | — | — | — | — | — | — |  |  |  |  |  |  |
| **CA** | |  |  |  |  |  |  |  |  |  |  |  |  |  |  |  |  |  |  |  |
| **CO** | |  |  |  |  |  |  |  |  |  |  |  |  |  |  |  |  |  |  |  |
| **CT** | |  |  |  |  |  |  |  |  |  |  |  |  |  |  |  |  |  |  |  |
| **DC** | | — | — | — | — | — | — | — | — | — | — | — | — | — |  |  |  |  |  |  |
| **DE** | | — | — | — | — | — | — | — | — | — | — | — | — | — |  |  |  |  |  |  |
| **FL** | |  |  |  |  |  |  |  |  |  |  |  |  |  |  |  |  |  |  |  |
| **GA** | |  |  |  |  |  |  |  |  |  |  |  |  |  |  |  |  |  |  |  |
| **HI** | |  |  |  |  |  |  |  |  |  |  |  |  |  |  |  |  |  |  |  |
| **IA** | |  |  |  |  |  |  |  |  |  |  |  |  |  |  |  |  |  |  |  |
| **ID** | |  |  |  |  |  |  |  |  |  |  |  |  |  |  |  |  |  |  |  |
| **IL** | |  |  |  |  |  |  |  |  |  |  |  |  |  |  |  |  |  |  |  |
| **IN** | |  |  |  |  |  |  |  |  |  |  |  |  |  |  |  |  |  |  |  |
| **KS** | |  |  |  |  |  |  |  |  |  |  |  |  |  |  |  |  |  |  |  |
| **KY** | |  |  |  |  |  |  |  |  |  |  |  |  |  |  |  |  |  |  |  |
| **LA** | |  |  |  |  |  |  |  |  |  |  |  |  |  |  |  |  |  |  |  |
| **MA** | |  |  |  |  |  |  |  |  |  |  |  |  |  |  |  |  |  |  |  |
| **MD** | |  |  |  |  |  |  |  |  |  |  |  |  |  |  |  |  |  |  |  |
| **ME** | |  |  |  |  |  |  |  |  |  |  |  |  |  |  |  |  |  |  |  |
| **MI** | |  |  |  |  |  |  |  |  |  |  |  |  |  |  |  |  |  |  |  |
| **MN** | |  |  |  |  |  |  |  |  |  |  |  |  |  |  |  |  |  |  |  |
| **MO** | |  |  |  |  |  |  |  |  |  |  |  |  |  |  |  |  |  |  |  |
| **MS** | |  |  |  |  |  |  |  |  |  |  |  |  |  |  |  |  |  |  |  |
| **MT** | |  |  |  |  |  |  |  |  |  |  |  |  |  |  |  |  |  |  |  |
| **NC** | |  |  |  |  |  |  |  |  |  |  |  |  |  |  |  |  |  |  |  |
| **ND** | |  |  |  |  |  |  |  |  |  |  |  |  |  |  |  |  |  |  |  |
| **NE** | |  |  |  |  |  |  |  |  |  |  |  |  |  |  |  |  |  |  |  |
| **NH** | |  |  |  |  |  |  |  |  |  |  |  |  |  |  |  |  |  |  |  |
| **NJ** | |  |  |  |  |  |  |  |  |  |  |  |  |  |  |  |  |  |  |  |
| **NM** | |  |  |  |  |  |  |  |  |  |  |  |  |  |  |  |  |  |  |  |
| **NV** | | — | — | — | — | — | — | — | — | — | — | — | — | — |  |  |  |  |  |  |
| **NY** | |  |  |  |  |  |  |  |  |  |  |  |  |  |  |  |  |  |  |  |
| **OH** | |  |  |  |  |  |  |  |  |  |  |  |  |  |  |  |  |  |  |  |
| **OK** | |  |  |  |  |  |  |  |  |  |  |  |  |  |  |  |  |  |  |  |
| **OR** | | — | — | — | — | — | — | — | — | — | — | — | — | — |  |  |  |  |  |  |
| **PA** | |  |  |  |  |  |  |  |  |  |  |  |  |  |  |  |  |  |  |  |
| **RI** | | — | — | — | — | — | — | — | — | — | — | — | — | — |  |  |  |  |  |  |
| **SC** | |  |  |  |  |  |  |  |  |  |  |  |  |  |  |  |  |  |  |  |
| **SD** | |  |  |  |  |  |  |  |  |  |  |  |  |  |  |  |  |  |  |  |
| **TN** | |  |  |  |  |  |  |  |  |  |  |  |  |  |  |  |  |  |  |  |
| **TX** | |  |  |  |  |  |  |  |  |  |  |  |  |  |  |  |  |  |  |  |
| **UT** | |  |  |  |  |  |  |  |  |  |  |  |  |  |  |  |  |  |  |  |
| **VA** | |  |  |  |  |  |  |  |  |  |  |  |  |  |  |  |  |  |  |  |
| **VT** | |  |  |  |  |  |  |  |  |  |  |  |  |  |  |  |  |  |  |  |
| **WA** | |  |  |  |  |  |  |  |  |  |  |  |  |  |  |  |  |  |  |  |
| **WI** | |  |  |  |  |  |  |  |  |  |  |  |  |  |  |  |  |  |  |  |
| **WV** | |  |  |  |  |  |  |  |  |  |  |  |  |  |  |  |  |  |  |  |
| **WY** | |  |  |  |  |  |  |  |  |  |  |  |  |  |  |  |  |  |  |  |
| **FFS** | **Mean** | 86.8 | 85.8 | 83.4 | 84.3 | 79.1 | 75.6 | 77.8 | 73.2 | 73.5 | 69.6 | 70.8 | 70.7 | 70.7 | 66.7 | 59.6 | 55.3 | 54.6 | 47.4 | 49.3 |
|  | **SD** | 9.5 | 12.3 | 14.0 | 13.1 | 18.5 | 24.2 | 23.2 | 26.3 | 28.0 | 31.3 | 27.3 | 27.0 | 28.1 | 24.7 | 28.6 | 29.0 | 29.7 | 29.5 | 29.8 |
| **CMC** | **Min** | 74.5 | 64.8 | 64.1 | 72.5 | 74.9 | 67.2 | 76.0 | 25.7 | 28.7 | 13.0 | 73.0 | 43.0 | 17.2 | 76.9 | 6.5 | 6.4 | 62.6 | 13.6 | 64.5 |
|  | **Max** | 91.9 | 91.2 | 91.2 | 93.6 | 93.7 | 93.6 | 94.1 | 93.9 | 93.4 | 94.4 | 94.0 | 93.0 | 92.5 | 93.9 | 94.3 | 93.8 | 93.3 | 92.7 | 93.1 |
| **LEGEND:** | | | | | | | | | | | | | | | | | | | | |
| **—** **Data not available** | | | | | | | | | | | | | | | | | | | | |
| **Low rates (<10%) of CMC enrollment** | | | | | | | | | | | | | | | | | | | | |
| **Low concern** | | | | | | | | | | | | | | | | | | | | |
| **Did not meet criteria** | | | | | | | | | | | | | | | | | | | | |

**Abbreviations:** CMC = comprehensive managed care; FFS = fee-for-service; SD = standard deviation

**Notes:** Measure was defined as the percentage of enrollees with any outpatient claim. State-years were identified as low concern if data for CMC enrollees were comparable to (i.e. within 2 standard deviations of) the national FFS population in the year on the measure.

# **Figure A6. CMC Results for Mean Number of Outpatient Claims per Enrollee**

| **STATE** | | **2001** | **2002** | **2003** | **2004** | **2005** | **2006** | **2007** | **2008** | **2009** | **2010** | **2011** | **2012** | **2013** | **2014** | **2015** | **2016** | **2017** | **2018** | **2019** |
| --- | --- | --- | --- | --- | --- | --- | --- | --- | --- | --- | --- | --- | --- | --- | --- | --- | --- | --- | --- | --- |
| **AK** | |  |  |  |  |  |  |  |  |  |  |  |  |  |  |  |  |  |  |  |
| **AL** | |  |  |  |  |  |  |  |  |  |  |  |  |  |  |  |  |  |  |  |
| **AR** | |  |  |  |  |  |  |  |  |  |  |  |  |  |  |  |  |  |  |  |
| **AZ** | | — | — | — | — | — | — | — | — | — | — | — | — | — |  |  |  |  |  |  |
| **CA** | |  |  |  |  |  |  |  |  |  |  |  |  |  |  |  |  |  |  |  |
| **CO** | |  |  |  |  |  |  |  |  |  |  |  |  |  |  |  |  |  |  |  |
| **CT** | |  |  |  |  |  |  |  |  |  |  |  |  |  |  |  |  |  |  |  |
| **DC** | | — | — | — | — | — | — | — | — | — | — | — | — | — |  |  |  |  |  |  |
| **DE** | | — | — | — | — | — | — | — | — | — | — | — | — | — |  |  |  |  |  |  |
| **FL** | |  |  |  |  |  |  |  |  |  |  |  |  |  |  |  |  |  |  |  |
| **GA** | |  |  |  |  |  |  |  |  |  |  |  |  |  |  |  |  |  |  |  |
| **HI** | |  |  |  |  |  |  |  |  |  |  |  |  |  |  |  |  |  |  |  |
| **IA** | |  |  |  |  |  |  |  |  |  |  |  |  |  |  |  |  |  |  |  |
| **ID** | |  |  |  |  |  |  |  |  |  |  |  |  |  |  |  |  |  |  |  |
| **IL** | |  |  |  |  |  |  |  |  |  |  |  |  |  |  |  |  |  |  |  |
| **IN** | |  |  |  |  |  |  |  |  |  |  |  |  |  |  |  |  |  |  |  |
| **KS** | |  |  |  |  |  |  |  |  |  |  |  |  |  |  |  |  |  |  |  |
| **KY** | |  |  |  |  |  |  |  |  |  |  |  |  |  |  |  |  |  |  |  |
| **LA** | |  |  |  |  |  |  |  |  |  |  |  |  |  |  |  |  |  |  |  |
| **MA** | |  |  |  |  |  |  |  |  |  |  |  |  |  |  |  |  |  |  |  |
| **MD** | |  |  |  |  |  |  |  |  |  |  |  |  |  |  |  |  |  |  |  |
| **ME** | |  |  |  |  |  |  |  |  |  |  |  |  |  |  |  |  |  |  |  |
| **MI** | |  |  |  |  |  |  |  |  |  |  |  |  |  |  |  |  |  |  |  |
| **MN** | |  |  |  |  |  |  |  |  |  |  |  |  |  |  |  |  |  |  |  |
| **MO** | |  |  |  |  |  |  |  |  |  |  |  |  |  |  |  |  |  |  |  |
| **MS** | |  |  |  |  |  |  |  |  |  |  |  |  |  |  |  |  |  |  |  |
| **MT** | |  |  |  |  |  |  |  |  |  |  |  |  |  |  |  |  |  |  |  |
| **NC** | |  |  |  |  |  |  |  |  |  |  |  |  |  |  |  |  |  |  |  |
| **ND** | |  |  |  |  |  |  |  |  |  |  |  |  |  |  |  |  |  |  |  |
| **NE** | |  |  |  |  |  |  |  |  |  |  |  |  |  |  |  |  |  |  |  |
| **NH** | |  |  |  |  |  |  |  |  |  |  |  |  |  |  |  |  |  |  |  |
| **NJ** | |  |  |  |  |  |  |  |  |  |  |  |  |  |  |  |  |  |  |  |
| **NM** | |  |  |  |  |  |  |  |  |  |  |  |  |  |  |  |  |  |  |  |
| **NV** | | — | — | — | — | — | — | — | — | — | — | — | — | — |  |  |  |  |  |  |
| **NY** | |  |  |  |  |  |  |  |  |  |  |  |  |  |  |  |  |  |  |  |
| **OH** | |  |  |  |  |  |  |  |  |  |  |  |  |  |  |  |  |  |  |  |
| **OK** | |  |  |  |  |  |  |  |  |  |  |  |  |  |  |  |  |  |  |  |
| **OR** | | — | — | — | — | — | — | — | — | — | — | — | — | — |  |  |  |  |  |  |
| **PA** | |  |  |  |  |  |  |  |  |  |  |  |  |  |  |  |  |  |  |  |
| **RI** | | — | — | — | — | — | — | — | — | — | — | — | — | — |  |  |  |  |  |  |
| **SC** | |  |  |  |  |  |  |  |  |  |  |  |  |  |  |  |  |  |  |  |
| **SD** | |  |  |  |  |  |  |  |  |  |  |  |  |  |  |  |  |  |  |  |
| **TN** | |  |  |  |  |  |  |  |  |  |  |  |  |  |  |  |  |  |  |  |
| **TX** | |  |  |  |  |  |  |  |  |  |  |  |  |  |  |  |  |  |  |  |
| **UT** | |  |  |  |  |  |  |  |  |  |  |  |  |  |  |  |  |  |  |  |
| **VA** | |  |  |  |  |  |  |  |  |  |  |  |  |  |  |  |  |  |  |  |
| **VT** | |  |  |  |  |  |  |  |  |  |  |  |  |  |  |  |  |  |  |  |
| **WA** | |  |  |  |  |  |  |  |  |  |  |  |  |  |  |  |  |  |  |  |
| **WI** | |  |  |  |  |  |  |  |  |  |  |  |  |  |  |  |  |  |  |  |
| **WV** | |  |  |  |  |  |  |  |  |  |  |  |  |  |  |  |  |  |  |  |
| **WY** | |  |  |  |  |  |  |  |  |  |  |  |  |  |  |  |  |  |  |  |
| **FFS** | **Mean** | 29.7 | 31.0 | 29.8 | 30.3 | 30.3 | 30.9 | 33.2 | 33.8 | 31.9 | 31.1 | 31.6 | 33.9 | 36.1 | 36.8 | 37.5 | 38.1 | 39.6 | 44.1 | 46.6 |
|  | **SD** | 8.5 | 10.8 | 10.2 | 11.1 | 12.1 | 13.4 | 14.7 | 14.6 | 13.6 | 16.0 | 16.2 | 18.0 | 25.6 | 28.6 | 20.2 | 18.9 | 25.7 | 49.2 | 41.1 |
| **CMC** | **Min** | 12.9 | 11.0 | 11.0 | 13.5 | 11.0 | 10.7 | 5.9 | 10.8 | 11.7 | 2.8 | 3.5 | 8.5 | 9.0 | 10.1 | 6.4 | 5.8 | 8.2 | 9.1 | 8.9 |
|  | **Max** | 24.0 | 25.0 | 29.4 | 29.2 | 38.7 | 39.3 | 39.8 | 39.7 | 47.1 | 55.9 | 54.3 | 60.4 | 53.0 | 61.1 | 60.0 | 60.8 | 65.1 | 63.1 | 63.9 |
| **LEGEND:** | | | | | | | | | | | | | | | | | | | | |
| **—** **Data not available** | | | | | | | | | | | | | | | | | | | | |
| **Low rates (<10%) of CMC enrollment** | | | | | | | | | | | | | | | | | | | | |
| **Low concern** | | | | | | | | | | | | | | | | | | | | |
| **Did not meet criteria** | | | | | | | | | | | | | | | | | | | | |

**Abbreviations:** CMC = comprehensive managed care; FFS = fee-for-service; SD = standard deviation

**Notes:** Measure was defined as the mean number of outpatient claims per enrollee with >1 outpatient claim. State-years were identified as low concern if data for CMC enrollees were comparable to (i.e. within 2 standard deviations of) the national FFS population in the year.

# **Figure A7. CMC Results for Percentage of Outpatient Claims with a Primary Diagnosis**

| **STATE** | | **2001** | **2002** | **2003** | **2004** | **2005** | **2006** | **2007** | **2008** | **2009** | **2010** | **2011** | **2012** | **2013** | **2014** | **2015** | **2016** | **2017** | **2018** | **2019** |
| --- | --- | --- | --- | --- | --- | --- | --- | --- | --- | --- | --- | --- | --- | --- | --- | --- | --- | --- | --- | --- |
| **AK** | |  |  |  |  |  |  |  |  |  |  |  |  |  |  |  |  |  |  |  |
| **AL** | |  |  |  |  |  |  |  |  |  |  |  |  |  |  |  |  |  |  |  |
| **AR** | |  |  |  |  |  |  |  |  |  |  |  |  |  |  |  |  |  |  |  |
| **AZ** | | — | — | — | — | — | — | — | — | — | — | — | — | — |  |  |  |  |  |  |
| **CA** | |  |  |  |  |  |  |  |  |  |  |  |  |  |  |  |  |  |  |  |
| **CO** | |  |  |  |  |  |  |  |  |  |  |  |  |  |  |  |  |  |  |  |
| **CT** | |  |  |  |  |  |  |  |  |  |  |  |  |  |  |  |  |  |  |  |
| **DC** | | — | — | — | — | — | — | — | — | — | — | — | — | — |  |  |  |  |  |  |
| **DE** | | — | — | — | — | — | — | — | — | — | — | — | — | — |  |  |  |  |  |  |
| **FL** | |  |  |  |  |  |  |  |  |  |  |  |  |  |  |  |  |  |  |  |
| **GA** | |  |  |  |  |  |  |  |  |  |  |  |  |  |  |  |  |  |  |  |
| **HI** | |  |  |  |  |  |  |  |  |  |  |  |  |  |  |  |  |  |  |  |
| **IA** | |  |  |  |  |  |  |  |  |  |  |  |  |  |  |  |  |  |  |  |
| **ID** | |  |  |  |  |  |  |  |  |  |  |  |  |  |  |  |  |  |  |  |
| **IL** | |  |  |  |  |  |  |  |  |  |  |  |  |  |  |  |  |  |  |  |
| **IN** | |  |  |  |  |  |  |  |  |  |  |  |  |  |  |  |  |  |  |  |
| **KS** | |  |  |  |  |  |  |  |  |  |  |  |  |  |  |  |  |  |  |  |
| **KY** | |  |  |  |  |  |  |  |  |  |  |  |  |  |  |  |  |  |  |  |
| **LA** | |  |  |  |  |  |  |  |  |  |  |  |  |  |  |  |  |  |  |  |
| **MA** | |  |  |  |  |  |  |  |  |  |  |  |  |  |  |  |  |  |  |  |
| **MD** | |  |  |  |  |  |  |  |  |  |  |  |  |  |  |  |  |  |  |  |
| **ME** | |  |  |  |  |  |  |  |  |  |  |  |  |  |  |  |  |  |  |  |
| **MI** | |  |  |  |  |  |  |  |  |  |  |  |  |  |  |  |  |  |  |  |
| **MN** | |  |  |  |  |  |  |  |  |  |  |  |  |  |  |  |  |  |  |  |
| **MO** | |  |  |  |  |  |  |  |  |  |  |  |  |  |  |  |  |  |  |  |
| **MS** | |  |  |  |  |  |  |  |  |  |  |  |  |  |  |  |  |  |  |  |
| **MT** | |  |  |  |  |  |  |  |  |  |  |  |  |  |  |  |  |  |  |  |
| **NC** | |  |  |  |  |  |  |  |  |  |  |  |  |  |  |  |  |  |  |  |
| **ND** | |  |  |  |  |  |  |  |  |  |  |  |  |  |  |  |  |  |  |  |
| **NE** | |  |  |  |  |  |  |  |  |  |  |  |  |  |  |  |  |  |  |  |
| **NH** | |  |  |  |  |  |  |  |  |  |  |  |  |  |  |  |  |  |  |  |
| **NJ** | |  |  |  |  |  |  |  |  |  |  |  |  |  |  |  |  |  |  |  |
| **NM** | |  |  |  |  |  |  |  |  |  |  |  |  |  |  |  |  |  |  |  |
| **NV** | | — | — | — | — | — | — | — | — | — | — | — | — | — |  |  |  |  |  |  |
| **NY** | |  |  |  |  |  |  |  |  |  |  |  |  |  |  |  |  |  |  |  |
| **OH** | |  |  |  |  |  |  |  |  |  |  |  |  |  |  |  |  |  |  |  |
| **OK** | |  |  |  |  |  |  |  |  |  |  |  |  |  |  |  |  |  |  |  |
| **OR** | | — | — | — | — | — | — | — | — | — | — | — | — | — |  |  |  |  |  |  |
| **PA** | |  |  |  |  |  |  |  |  |  |  |  |  |  |  |  |  |  |  |  |
| **RI** | | — | — | — | — | — | — | — | — | — | — | — | — | — |  |  |  |  |  |  |
| **SC** | |  |  |  |  |  |  |  |  |  |  |  |  |  |  |  |  |  |  |  |
| **SD** | |  |  |  |  |  |  |  |  |  |  |  |  |  |  |  |  |  |  |  |
| **TN** | |  |  |  |  |  |  |  |  |  |  |  |  |  |  |  |  |  |  |  |
| **TX** | |  |  |  |  |  |  |  |  |  |  |  |  |  |  |  |  |  |  |  |
| **UT** | |  |  |  |  |  |  |  |  |  |  |  |  |  |  |  |  |  |  |  |
| **VA** | |  |  |  |  |  |  |  |  |  |  |  |  |  |  |  |  |  |  |  |
| **VT** | |  |  |  |  |  |  |  |  |  |  |  |  |  |  |  |  |  |  |  |
| **WA** | |  |  |  |  |  |  |  |  |  |  |  |  |  |  |  |  |  |  |  |
| **WI** | |  |  |  |  |  |  |  |  |  |  |  |  |  |  |  |  |  |  |  |
| **WV** | |  |  |  |  |  |  |  |  |  |  |  |  |  |  |  |  |  |  |  |
| **WY** | |  |  |  |  |  |  |  |  |  |  |  |  |  |  |  |  |  |  |  |
| **FFS** | **Mean** | 99.4 | 99.5 | 99.4 | 99.5 | 99.6 | 99.8 | 99.8 | 99.6 | 99.3 | 99.2 | 99.2 | 99.2 | 97.8 | 98.2 | 98.1 | 98.2 | 98.6 | 99.6 | 99.7 |
|  | **SD** | 1.1 | 1.0 | 1.4 | 1.3 | 1.0 | 0.7 | 0.7 | 1.4 | 2.1 | 2.6 | 2.8 | 2.9 | 10.3 | 7.3 | 9.4 | 9.8 | 6.5 | 1.2 | 1.1 |
| **CMC** | **Min** | 98.3 | 99.0 | 98.8 | 98.5 | 99.0 | 98.4 | 99.3 | 97.6 | 95.1 | 94.7 | 94.3 | 98.5 | 91.9 | 90.8 | 90.2 | 80.8 | 87.2 | 98.0 | 98.3 |
|  | **Max** | 100 | 100 | 100 | 100 | 100 | 100 | 100 | 100 | 100 | 100 | 100 | 100 | 100 | 100 | 100 | 100 | 100 | 100 | 100 |
| **LEGEND:** | | | | | | | | | | | | | | | | | | | | |
| **—** **Data not available** | | | | | | | | | | | | | | | | | | | | |
| **Low rates (<10%) of CMC enrollment** | | | | | | | | | | | | | | | | | | | | |
| **Low concern** | | | | | | | | | | | | | | | | | | | | |
| **Did not meet criteria** | | | | | | | | | | | | | | | | | | | | |

**Abbreviations:** CMC = comprehensive managed care; FFS = fee-for-service; SD = standard deviation

**Notes:** Measure was defined as the percentage of outpatient claims with a non-missing value in the first diagnosis code field, considering fields with all zeros, nines, dots, or spaces as missing. State-years were identified as low concern if data for CMC enrollees were comparable to (i.e. within 2 standard deviations of) the national FFS population in the year.

# **Figure A8. CMC Results for Percentage of Outpatient Claims with a Procedure Code**

| **STATE** | | **2001** | **2002** | **2003** | **2004** | **2005** | **2006** | **2007** | **2008** | **2009** | **2010** | **2011** | **2012** | **2013** | **2014** | **2015** | **2016** | **2017** | **2018** | **2019** |
| --- | --- | --- | --- | --- | --- | --- | --- | --- | --- | --- | --- | --- | --- | --- | --- | --- | --- | --- | --- | --- |
| **AK** | |  |  |  |  |  |  |  |  |  |  |  |  |  |  |  |  |  |  |  |
| **AL** | |  |  |  |  |  |  |  |  |  |  |  |  |  |  |  |  |  |  |  |
| **AR** | |  |  |  |  |  |  |  |  |  |  |  |  |  |  |  |  |  |  |  |
| **AZ** | | — | — | — | — | — | — | — | — | — | — | — | — | — |  |  |  |  |  |  |
| **CA** | |  |  |  |  |  |  |  |  |  |  |  |  |  |  |  |  |  |  |  |
| **CO** | |  |  |  |  |  |  |  |  |  |  |  |  |  |  |  |  |  |  |  |
| **CT** | |  |  |  |  |  |  |  |  |  |  |  |  |  |  |  |  |  |  |  |
| **DC** | | — | — | — | — | — | — | — | — | — | — | — | — | — |  |  |  |  |  |  |
| **DE** | | — | — | — | — | — | — | — | — | — | — | — | — | — |  |  |  |  |  |  |
| **FL** | |  |  |  |  |  |  |  |  |  |  |  |  |  |  |  |  |  |  |  |
| **GA** | |  |  |  |  |  |  |  |  |  |  |  |  |  |  |  |  |  |  |  |
| **HI** | |  |  |  |  |  |  |  |  |  |  |  |  |  |  |  |  |  |  |  |
| **IA** | |  |  |  |  |  |  |  |  |  |  |  |  |  |  |  |  |  |  |  |
| **ID** | |  |  |  |  |  |  |  |  |  |  |  |  |  |  |  |  |  |  |  |
| **IL** | |  |  |  |  |  |  |  |  |  |  |  |  |  |  |  |  |  |  |  |
| **IN** | |  |  |  |  |  |  |  |  |  |  |  |  |  |  |  |  |  |  |  |
| **KS** | |  |  |  |  |  |  |  |  |  |  |  |  |  |  |  |  |  |  |  |
| **KY** | |  |  |  |  |  |  |  |  |  |  |  |  |  |  |  |  |  |  |  |
| **LA** | |  |  |  |  |  |  |  |  |  |  |  |  |  |  |  |  |  |  |  |
| **MA** | |  |  |  |  |  |  |  |  |  |  |  |  |  |  |  |  |  |  |  |
| **MD** | |  |  |  |  |  |  |  |  |  |  |  |  |  |  |  |  |  |  |  |
| **ME** | |  |  |  |  |  |  |  |  |  |  |  |  |  |  |  |  |  |  |  |
| **MI** | |  |  |  |  |  |  |  |  |  |  |  |  |  |  |  |  |  |  |  |
| **MN** | |  |  |  |  |  |  |  |  |  |  |  |  |  |  |  |  |  |  |  |
| **MO** | |  |  |  |  |  |  |  |  |  |  |  |  |  |  |  |  |  |  |  |
| **MS** | |  |  |  |  |  |  |  |  |  |  |  |  |  |  |  |  |  |  |  |
| **MT** | |  |  |  |  |  |  |  |  |  |  |  |  |  |  |  |  |  |  |  |
| **NC** | |  |  |  |  |  |  |  |  |  |  |  |  |  |  |  |  |  |  |  |
| **ND** | |  |  |  |  |  |  |  |  |  |  |  |  |  |  |  |  |  |  |  |
| **NE** | |  |  |  |  |  |  |  |  |  |  |  |  |  |  |  |  |  |  |  |
| **NH** | |  |  |  |  |  |  |  |  |  |  |  |  |  |  |  |  |  |  |  |
| **NJ** | |  |  |  |  |  |  |  |  |  |  |  |  |  |  |  |  |  |  |  |
| **NM** | |  |  |  |  |  |  |  |  |  |  |  |  |  |  |  |  |  |  |  |
| **NV** | | — | — | — | — | — | — | — | — | — | — | — | — | — |  |  |  |  |  |  |
| **NY** | |  |  |  |  |  |  |  |  |  |  |  |  |  |  |  |  |  |  |  |
| **OH** | |  |  |  |  |  |  |  |  |  |  |  |  |  |  |  |  |  |  |  |
| **OK** | |  |  |  |  |  |  |  |  |  |  |  |  |  |  |  |  |  |  |  |
| **OR** | | — | — | — | — | — | — | — | — | — | — | — | — | — |  |  |  |  |  |  |
| **PA** | |  |  |  |  |  |  |  |  |  |  |  |  |  |  |  |  |  |  |  |
| **RI** | | — | — | — | — | — | — | — | — | — | — | — | — | — |  |  |  |  |  |  |
| **SC** | |  |  |  |  |  |  |  |  |  |  |  |  |  |  |  |  |  |  |  |
| **SD** | |  |  |  |  |  |  |  |  |  |  |  |  |  |  |  |  |  |  |  |
| **TN** | |  |  |  |  |  |  |  |  |  |  |  |  |  |  |  |  |  |  |  |
| **TX** | |  |  |  |  |  |  |  |  |  |  |  |  |  |  |  |  |  |  |  |
| **UT** | |  |  |  |  |  |  |  |  |  |  |  |  |  |  |  |  |  |  |  |
| **VA** | |  |  |  |  |  |  |  |  |  |  |  |  |  |  |  |  |  |  |  |
| **VT** | |  |  |  |  |  |  |  |  |  |  |  |  |  |  |  |  |  |  |  |
| **WA** | |  |  |  |  |  |  |  |  |  |  |  |  |  |  |  |  |  |  |  |
| **WI** | |  |  |  |  |  |  |  |  |  |  |  |  |  |  |  |  |  |  |  |
| **WV** | |  |  |  |  |  |  |  |  |  |  |  |  |  |  |  |  |  |  |  |
| **WY** | |  |  |  |  |  |  |  |  |  |  |  |  |  |  |  |  |  |  |  |
| **FFS** | **Mean** | 98.5 | 98.5 | 98.4 | 98.4 | 98.2 | 98.3 | 98.2 | 98.1 | 95.9 | 97.8 | 98.1 | 97.5 | 96.8 | 91.6 | 86.6 | 81.9 | 83.0 | 85.5 | 88.1 |
|  | **SD** | 4.0 | 4.2 | 3.9 | 3.9 | 4.5 | 4.1 | 4.5 | 4.4 | 14.2 | 6.4 | 4.1 | 7.9 | 7.6 | 13.8 | 18.4 | 22.3 | 22.3 | 20.5 | 15.4 |
| **CMC** | **Min** | 96.1 | 94.0 | 92.8 | 92.8 | 90.6 | 91.0 | 89.3 | 89.4 | 81.4 | 85.3 | 92.1 | 90.6 | 89.8 | 72.2 | 54.6 | 43.5 | 45.3 | 46.9 | 65.5 |
|  | **Max** | 100 | 100 | 100 | 100 | 100 | 100 | 100 | 100 | 100 | 100 | 100 | 100 | 100 | 100 | 100 | 100 | 100 | 100 | 100 |
| **LEGEND:** | | | | | | | | | | | | | | | | | | | | |
| **—** **Data not available** | | | | | | | | | | | | | | | | | | | | |
| **Low rates (<10%) of CMC enrollment** | | | | | | | | | | | | | | | | | | | | |
| **Low concern** | | | | | | | | | | | | | | | | | | | | |
| **Did not meet criteria** | | | | | | | | | | | | | | | | | | | | |

**Abbreviations:** CMC = comprehensive managed care; FFS = fee-for-service; SD = standard deviation

**Notes:** Measure was defined as the percentage of outpatient claims with a non-missing value in the first procedure code field, considering fields with all zeros, nines, dots, or spaces as missing. State-years were identified as low concern if data for CMC enrollees were comparable to (i.e. within 2 standard deviations of) the national FFS population in the year.

# **Figure A9. CMC Results for Percentage of Enrollees with Any Pharmacy Claim**

| **STATE** | | **2001** | **2002** | **2003** | **2004** | **2005** | **2006** | **2007** | **2008** | **2009** | **2010** | **2011** | **2012** | **2013** | **2014** | **2015** | **2016** | **2017** | **2018** | **2019** |
| --- | --- | --- | --- | --- | --- | --- | --- | --- | --- | --- | --- | --- | --- | --- | --- | --- | --- | --- | --- | --- |
| **AK** | |  |  |  |  |  |  |  |  |  |  |  |  |  |  |  |  |  |  |  |
| **AL** | |  |  |  |  |  |  |  |  |  |  |  |  |  |  |  |  |  |  |  |
| **AR** | |  |  |  |  |  |  |  |  |  |  |  |  |  |  |  |  |  |  |  |
| **AZ** | | — | — | — | — | — | — | — | — | — | — | — | — | — |  |  |  |  |  |  |
| **CA** | |  |  |  |  |  |  |  |  |  |  |  |  |  |  |  |  |  |  |  |
| **CO** | |  |  |  |  |  |  |  |  |  |  |  |  |  |  |  |  |  |  |  |
| **CT** | |  |  |  |  |  |  |  |  |  |  |  |  |  |  |  |  |  |  |  |
| **DC** | | — | — | — | — | — | — | — | — | — | — | — | — | — |  |  |  |  |  |  |
| **DE** | | — | — | — | — | — | — | — | — | — | — | — | — | — |  |  |  |  |  |  |
| **FL** | |  |  |  |  |  |  |  |  |  |  |  |  |  |  |  |  |  |  |  |
| **GA** | |  |  |  |  |  |  |  |  |  |  |  |  |  |  |  |  |  |  |  |
| **HI** | |  |  |  |  |  |  |  |  |  |  |  |  |  |  |  |  |  |  |  |
| **IA** | |  |  |  |  |  |  |  |  |  |  |  |  |  |  |  |  |  |  |  |
| **ID** | |  |  |  |  |  |  |  |  |  |  |  |  |  |  |  |  |  |  |  |
| **IL** | |  |  |  |  |  |  |  |  |  |  |  |  |  |  |  |  |  |  |  |
| **IN** | |  |  |  |  |  |  |  |  |  |  |  |  |  |  |  |  |  |  |  |
| **KS** | |  |  |  |  |  |  |  |  |  |  |  |  |  |  |  |  |  |  |  |
| **KY** | |  |  |  |  |  |  |  |  |  |  |  |  |  |  |  |  |  |  |  |
| **LA** | |  |  |  |  |  |  |  |  |  |  |  |  |  |  |  |  |  |  |  |
| **MA** | |  |  |  |  |  |  |  |  |  |  |  |  |  |  |  |  |  |  |  |
| **MD** | |  |  |  |  |  |  |  |  |  |  |  |  |  |  |  |  |  |  |  |
| **ME** | |  |  |  |  |  |  |  |  |  |  |  |  |  |  |  |  |  |  |  |
| **MI** | |  |  |  |  |  |  |  |  |  |  |  |  |  |  |  |  |  |  |  |
| **MN** | |  |  |  |  |  |  |  |  |  |  |  |  |  |  |  |  |  |  |  |
| **MO** | |  |  |  |  |  |  |  |  |  |  |  |  |  |  |  |  |  |  |  |
| **MS** | |  |  |  |  |  |  |  |  |  |  |  |  |  |  |  |  |  |  |  |
| **MT** | |  |  |  |  |  |  |  |  |  |  |  |  |  |  |  |  |  |  |  |
| **NC** | |  |  |  |  |  |  |  |  |  |  |  |  |  |  |  |  |  |  |  |
| **ND** | |  |  |  |  |  |  |  |  |  |  |  |  |  |  |  |  |  |  |  |
| **NE** | |  |  |  |  |  |  |  |  |  |  |  |  |  |  |  |  |  |  |  |
| **NH** | |  |  |  |  |  |  |  |  |  |  |  |  |  |  |  |  |  |  |  |
| **NJ** | |  |  |  |  |  |  |  |  |  |  |  |  |  |  |  |  |  |  |  |
| **NM** | |  |  |  |  |  |  |  |  |  |  |  |  |  |  |  |  |  |  |  |
| **NV** | | — | — | — | — | — | — | — | — | — | — | — | — | — |  |  |  |  |  |  |
| **NY** | |  |  |  |  |  |  |  |  |  |  |  |  |  |  |  |  |  |  |  |
| **OH** | |  |  |  |  |  |  |  |  |  |  |  |  |  |  |  |  |  |  |  |
| **OK** | |  |  |  |  |  |  |  |  |  |  |  |  |  |  |  |  |  |  |  |
| **OR** | | — | — | — | — | — | — | — | — | — | — | — | — | — |  |  |  |  |  |  |
| **PA** | |  |  |  |  |  |  |  |  |  |  |  |  |  |  |  |  |  |  |  |
| **RI** | | — | — | — | — | — | — | — | — | — | — | — | — | — |  |  |  |  |  |  |
| **SC** | |  |  |  |  |  |  |  |  |  |  |  |  |  |  |  |  |  |  |  |
| **SD** | |  |  |  |  |  |  |  |  |  |  |  |  |  |  |  |  |  |  |  |
| **TN** | |  |  |  |  |  |  |  |  |  |  |  |  |  |  |  |  |  |  |  |
| **TX** | |  |  |  |  |  |  |  |  |  |  |  |  |  |  |  |  |  |  |  |
| **UT** | |  |  |  |  |  |  |  |  |  |  |  |  |  |  |  |  |  |  |  |
| **VA** | |  |  |  |  |  |  |  |  |  |  |  |  |  |  |  |  |  |  |  |
| **VT** | |  |  |  |  |  |  |  |  |  |  |  |  |  |  |  |  |  |  |  |
| **WA** | |  |  |  |  |  |  |  |  |  |  |  |  |  |  |  |  |  |  |  |
| **WI** | |  |  |  |  |  |  |  |  |  |  |  |  |  |  |  |  |  |  |  |
| **WV** | |  |  |  |  |  |  |  |  |  |  |  |  |  |  |  |  |  |  |  |
| **WY** | |  |  |  |  |  |  |  |  |  |  |  |  |  |  |  |  |  |  |  |
| **FFS** | **Mean** | 83.7 | 83.9 | 80.7 | 82.5 | 80.1 | 76.6 | 75.6 | 72.4 | 74.0 | 70.3 | 70.2 | 66.8 | 66.5 | 56.6 | 50.8 | 41.7 | 42.2 | 37.2 | 38.3 |
|  | **SD** | 13.6 | 14.6 | 15.7 | 16.6 | 14.6 | 22.3 | 24.8 | 26.2 | 24.6 | 29.7 | 27.0 | 30.9 | 32.3 | 30.5 | 31.3 | 29.8 | 31.7 | 32.2 | 31.8 |
| **CMC** | **Min** | 71.5 | 70.6 | 59.1 | 74.7 | 73.4 | 44.4 | 42.3 | 80.0 | 78.9 | 77.8 | 70.5 | 15.1 | 75.0 | <0.1 | 2.3 | 0.0 | 0.0 | 0.0 | 0.0 |
|  | **Max** | 92.1 | 91.6 | 91.8 | 92.3 | 92.6 | 92.2 | 93.4 | 92.1 | 92.0 | 91.9 | 92.1 | 93.9 | 92.1 | 91.3 | 91.1 | 90.3 | 89.1 | 90.0 | 89.7 |
| **LEGEND:** | | | | | | | | | | | | | | | | | | | | |
| **—** **Data not available** | | | | | | | | | | | | | | | | | | | | |
| **Low rates (<10%) of CMC enrollment** | | | | | | | | | | | | | | | | | | | | |
| **Low concern** | | | | | | | | | | | | | | | | | | | | |
| **Did not meet criteria** | | | | | | | | | | | | | | | | | | | | |

**Abbreviations:** CMC = comprehensive managed care; FFS = fee-for-service; SD = standard deviation

**Notes:** Measure was defined as the percentage of enrollees with any pharmacy claim. State-years were identified as low concern if data for CMC enrollees were comparable to (i.e. within 2 standard deviations of) the national FFS population in the year.

# **Figure A10. CMC Results for Mean Number of Pharmacy Claims per Enrollee**

| **STATE** | | **2001** | **2002** | **2003** | **2004** | **2005** | **2006** | **2007** | **2008** | **2009** | **2010** | **2011** | **2012** | **2013** | **2014** | **2015** | **2016** | **2017** | **2018** | **2019** |
| --- | --- | --- | --- | --- | --- | --- | --- | --- | --- | --- | --- | --- | --- | --- | --- | --- | --- | --- | --- | --- |
| **AK** | |  |  |  |  |  |  |  |  |  |  |  |  |  |  |  |  |  |  |  |
| **AL** | |  |  |  |  |  |  |  |  |  |  |  |  |  |  |  |  |  |  |  |
| **AR** | |  |  |  |  |  |  |  |  |  |  |  |  |  |  |  |  |  |  |  |
| **AZ** | | — | — | — | — | — | — | — | — | — | — | — | — | — |  |  |  |  |  |  |
| **CA** | |  |  |  |  |  |  |  |  |  |  |  |  |  |  |  |  |  |  |  |
| **CO** | |  |  |  |  |  |  |  |  |  |  |  |  |  |  |  |  |  |  |  |
| **CT** | |  |  |  |  |  |  |  |  |  |  |  |  |  |  |  |  |  |  |  |
| **DC** | | — | — | — | — | — | — | — | — | — | — | — | — | — |  |  |  |  |  |  |
| **DE** | | — | — | — | — | — | — | — | — | — | — | — | — | — |  |  |  |  |  |  |
| **FL** | |  |  |  |  |  |  |  |  |  |  |  |  |  |  |  |  |  |  |  |
| **GA** | |  |  |  |  |  |  |  |  |  |  |  |  |  |  |  |  |  |  |  |
| **HI** | |  |  |  |  |  |  |  |  |  |  |  |  |  |  |  |  |  |  |  |
| **IA** | |  |  |  |  |  |  |  |  |  |  |  |  |  |  |  |  |  |  |  |
| **ID** | |  |  |  |  |  |  |  |  |  |  |  |  |  |  |  |  |  |  |  |
| **IL** | |  |  |  |  |  |  |  |  |  |  |  |  |  |  |  |  |  |  |  |
| **IN** | |  |  |  |  |  |  |  |  |  |  |  |  |  |  |  |  |  |  |  |
| **KS** | |  |  |  |  |  |  |  |  |  |  |  |  |  |  |  |  |  |  |  |
| **KY** | |  |  |  |  |  |  |  |  |  |  |  |  |  |  |  |  |  |  |  |
| **LA** | |  |  |  |  |  |  |  |  |  |  |  |  |  |  |  |  |  |  |  |
| **MA** | |  |  |  |  |  |  |  |  |  |  |  |  |  |  |  |  |  |  |  |
| **MD** | |  |  |  |  |  |  |  |  |  |  |  |  |  |  |  |  |  |  |  |
| **ME** | |  |  |  |  |  |  |  |  |  |  |  |  |  |  |  |  |  |  |  |
| **MI** | |  |  |  |  |  |  |  |  |  |  |  |  |  |  |  |  |  |  |  |
| **MN** | |  |  |  |  |  |  |  |  |  |  |  |  |  |  |  |  |  |  |  |
| **MO** | |  |  |  |  |  |  |  |  |  |  |  |  |  |  |  |  |  |  |  |
| **MS** | |  |  |  |  |  |  |  |  |  |  |  |  |  |  |  |  |  |  |  |
| **MT** | |  |  |  |  |  |  |  |  |  |  |  |  |  |  |  |  |  |  |  |
| **NC** | |  |  |  |  |  |  |  |  |  |  |  |  |  |  |  |  |  |  |  |
| **ND** | |  |  |  |  |  |  |  |  |  |  |  |  |  |  |  |  |  |  |  |
| **NE** | |  |  |  |  |  |  |  |  |  |  |  |  |  |  |  |  |  |  |  |
| **NH** | |  |  |  |  |  |  |  |  |  |  |  |  |  |  |  |  |  |  |  |
| **NJ** | |  |  |  |  |  |  |  |  |  |  |  |  |  |  |  |  |  |  |  |
| **NM** | |  |  |  |  |  |  |  |  |  |  |  |  |  |  |  |  |  |  |  |
| **NV** | | — | — | — | — | — | — | — | — | — | — | — | — | — |  |  |  |  |  |  |
| **NY** | |  |  |  |  |  |  |  |  |  |  |  |  |  |  |  |  |  |  |  |
| **OH** | |  |  |  |  |  |  |  |  |  |  |  |  |  |  |  |  |  |  |  |
| **OK** | |  |  |  |  |  |  |  |  |  |  |  |  |  |  |  |  |  |  |  |
| **OR** | | — | — | — | — | — | — | — | — | — | — | — | — | — |  |  |  |  |  |  |
| **PA** | |  |  |  |  |  |  |  |  |  |  |  |  |  |  |  |  |  |  |  |
| **RI** | | — | — | — | — | — | — | — | — | — | — | — | — | — |  |  |  |  |  |  |
| **SC** | |  |  |  |  |  |  |  |  |  |  |  |  |  |  |  |  |  |  |  |
| **SD** | |  |  |  |  |  |  |  |  |  |  |  |  |  |  |  |  |  |  |  |
| **TN** | |  |  |  |  |  |  |  |  |  |  |  |  |  |  |  |  |  |  |  |
| **TX** | |  |  |  |  |  |  |  |  |  |  |  |  |  |  |  |  |  |  |  |
| **UT** | |  |  |  |  |  |  |  |  |  |  |  |  |  |  |  |  |  |  |  |
| **VA** | |  |  |  |  |  |  |  |  |  |  |  |  |  |  |  |  |  |  |  |
| **VT** | |  |  |  |  |  |  |  |  |  |  |  |  |  |  |  |  |  |  |  |
| **WA** | |  |  |  |  |  |  |  |  |  |  |  |  |  |  |  |  |  |  |  |
| **WI** | |  |  |  |  |  |  |  |  |  |  |  |  |  |  |  |  |  |  |  |
| **WV** | |  |  |  |  |  |  |  |  |  |  |  |  |  |  |  |  |  |  |  |
| **WY** | |  |  |  |  |  |  |  |  |  |  |  |  |  |  |  |  |  |  |  |
| **FFS** | **Mean** | 44.6 | 44.9 | 44.1 | 46.1 | 49.8 | 49.0 | 53.2 | 53.0 | 56.0 | 53.6 | 53.1 | 52.9 | 51.1 | 51.6 | 58.4 | 45.0 | 48.0 | 43.6 | 42.1 |
|  | **SD** | 13.9 | 18.3 | 14.2 | 16.0 | 20.2 | 20.4 | 19.1 | 23.6 | 25.1 | 27.3 | 24.9 | 22.2 | 25.0 | 31.5 | 89.4 | 33.2 | 53.0 | 46.1 | 31.6 |
| **CMC** | **Min** | 16.9 | 14.1 | 16.0 | 14.8 | 15.7 | 15.7 | 17.5 | 10.2 | 17.4 | 2.6 | 4.3 | 19.3 | 9.0 | 2.0 | 2.9 | 2.9 | 4.9 | 5.6 | 5.5 |
|  | **Max** | 44.6 | 40.7 | 38.7 | 41.2 | 51.1 | 42.8 | 45.2 | 43.3 | 45.0 | 63.3 | 47.4 | 49.1 | 54.2 | 58.1 | 72.2 | 52.0 | 49.7 | 49.7 | 48.1 |
| **LEGEND:** | | | | | | | | | | | | | | | | | | | | |
| **—** **Data not available** | | | | | | | | | | | | | | | | | | | | |
| **Low rates (<10%) of CMC enrollment** | | | | | | | | | | | | | | | | | | | | |
| **Low concern** | | | | | | | | | | | | | | | | | | | | |
| **Did not meet criteria** | | | | | | | | | | | | | | | | | | | | |

**Abbreviations:** CMC = comprehensive managed care; FFS = fee-for-service; SD = standard deviation

**Notes:** Measure was defined as the mean number of pharmacy claims per enrollee with >1 pharmacy claim. State-years were identified as low concern if data for CMC enrollees were comparable to (i.e. within 2 standard deviations of) the national FFS population in the year.

# **Figure A11. CMC Results for Percentage of Pharmacy Claims with a Fill Date**

| **STATE** | | **2001** | **2002** | **2003** | **2004** | **2005** | **2006** | **2007** | **2008** | **2009** | **2010** | **2011** | **2012** | **2013** | **2014** | **2015** | **2016** | **2017** | **2018** | **2019** |
| --- | --- | --- | --- | --- | --- | --- | --- | --- | --- | --- | --- | --- | --- | --- | --- | --- | --- | --- | --- | --- |
| **AK** | |  |  |  |  |  |  |  |  |  |  |  |  |  |  |  |  |  |  |  |
| **AL** | |  |  |  |  |  |  |  |  |  |  |  |  |  |  |  |  |  |  |  |
| **AR** | |  |  |  |  |  |  |  |  |  |  |  |  |  |  |  |  |  |  |  |
| **AZ** | | — | — | — | — | — | — | — | — | — | — | — | — | — |  |  |  |  |  |  |
| **CA** | |  |  |  |  |  |  |  |  |  |  |  |  |  |  |  |  |  |  |  |
| **CO** | |  |  |  |  |  |  |  |  |  |  |  |  |  |  |  |  |  |  |  |
| **CT** | |  |  |  |  |  |  |  |  |  |  |  |  |  |  |  |  |  |  |  |
| **DC** | | — | — | — | — | — | — | — | — | — | — | — | — | — |  |  |  |  |  |  |
| **DE** | | — | — | — | — | — | — | — | — | — | — | — | — | — |  |  |  |  |  |  |
| **FL** | |  |  |  |  |  |  |  |  |  |  |  |  |  |  |  |  |  |  |  |
| **GA** | |  |  |  |  |  |  |  |  |  |  |  |  |  |  |  |  |  |  |  |
| **HI** | |  |  |  |  |  |  |  |  |  |  |  |  |  |  |  |  |  |  |  |
| **IA** | |  |  |  |  |  |  |  |  |  |  |  |  |  |  |  |  |  |  |  |
| **ID** | |  |  |  |  |  |  |  |  |  |  |  |  |  |  |  |  |  |  |  |
| **IL** | |  |  |  |  |  |  |  |  |  |  |  |  |  |  |  |  |  |  |  |
| **IN** | |  |  |  |  |  |  |  |  |  |  |  |  |  |  |  |  |  |  |  |
| **KS** | |  |  |  |  |  |  |  |  |  |  |  |  |  |  |  |  |  |  |  |
| **KY** | |  |  |  |  |  |  |  |  |  |  |  |  |  |  |  |  |  |  |  |
| **LA** | |  |  |  |  |  |  |  |  |  |  |  |  |  |  |  |  |  |  |  |
| **MA** | |  |  |  |  |  |  |  |  |  |  |  |  |  |  |  |  |  |  |  |
| **MD** | |  |  |  |  |  |  |  |  |  |  |  |  |  |  |  |  |  |  |  |
| **ME** | |  |  |  |  |  |  |  |  |  |  |  |  |  |  |  |  |  |  |  |
| **MI** | |  |  |  |  |  |  |  |  |  |  |  |  |  |  |  |  |  |  |  |
| **MN** | |  |  |  |  |  |  |  |  |  |  |  |  |  |  |  |  |  |  |  |
| **MO** | |  |  |  |  |  |  |  |  |  |  |  |  |  |  |  |  |  |  |  |
| **MS** | |  |  |  |  |  |  |  |  |  |  |  |  |  |  |  |  |  |  |  |
| **MT** | |  |  |  |  |  |  |  |  |  |  |  |  |  |  |  |  |  |  |  |
| **NC** | |  |  |  |  |  |  |  |  |  |  |  |  |  |  |  |  |  |  |  |
| **ND** | |  |  |  |  |  |  |  |  |  |  |  |  |  |  |  |  |  |  |  |
| **NE** | |  |  |  |  |  |  |  |  |  |  |  |  |  |  |  |  |  |  |  |
| **NH** | |  |  |  |  |  |  |  |  |  |  |  |  |  |  |  |  |  |  |  |
| **NJ** | |  |  |  |  |  |  |  |  |  |  |  |  |  |  |  |  |  |  |  |
| **NM** | |  |  |  |  |  |  |  |  |  |  |  |  |  |  |  |  |  |  |  |
| **NV** | | — | — | — | — | — | — | — | — | — | — | — | — | — |  |  |  |  |  |  |
| **NY** | |  |  |  |  |  |  |  |  |  |  |  |  |  |  |  |  |  |  |  |
| **OH** | |  |  |  |  |  |  |  |  |  |  |  |  |  |  |  |  |  |  |  |
| **OK** | |  |  |  |  |  |  |  |  |  |  |  |  |  |  |  |  |  |  |  |
| **OR** | | — | — | — | — | — | — | — | — | — | — | — | — | — |  |  |  |  |  |  |
| **PA** | |  |  |  |  |  |  |  |  |  |  |  |  |  |  |  |  |  |  |  |
| **RI** | | — | — | — | — | — | — | — | — | — | — | — | — | — |  |  |  |  |  |  |
| **SC** | |  |  |  |  |  |  |  |  |  |  |  |  |  |  |  |  |  |  |  |
| **SD** | |  |  |  |  |  |  |  |  |  |  |  |  |  |  |  |  |  |  |  |
| **TN** | |  |  |  |  |  |  |  |  |  |  |  |  |  |  |  |  |  |  |  |
| **TX** | |  |  |  |  |  |  |  |  |  |  |  |  |  |  |  |  |  |  |  |
| **UT** | |  |  |  |  |  |  |  |  |  |  |  |  |  |  |  |  |  |  |  |
| **VA** | |  |  |  |  |  |  |  |  |  |  |  |  |  |  |  |  |  |  |  |
| **VT** | |  |  |  |  |  |  |  |  |  |  |  |  |  |  |  |  |  |  |  |
| **WA** | |  |  |  |  |  |  |  |  |  |  |  |  |  |  |  |  |  |  |  |
| **WI** | |  |  |  |  |  |  |  |  |  |  |  |  |  |  |  |  |  |  |  |
| **WV** | |  |  |  |  |  |  |  |  |  |  |  |  |  |  |  |  |  |  |  |
| **WY** | |  |  |  |  |  |  |  |  |  |  |  |  |  |  |  |  |  |  |  |
| **FFS** | **Mean** | 100 | 100 | 100 | 100 | 100 | 100 | 100 | 100 | 100 | 100 | 100 | 100 | 100 | 100 | 100 | 100 | 100 | 100 | 100 |
|  | **SD** | 0 | 0 | 0 | 0 | 0 | 0 | 0 | 0 | 0 | 0 | 0 | 0 | 0 | 0 | 0 | 0 | 0 | 0 | 0 |
| **CMC** | **Min** | 100 | 100 | 100 | 100 | 100 | 100 | 100 | 100 | 100 | 100 | 100 | 100 | 100 | 100 | 100 | 100 | 100 | 100 | 100 |
|  | **Max** | 100 | 100 | 100 | 100 | 100 | 100 | 100 | 100 | 100 | 100 | 100 | 100 | 100 | 100 | 100 | 100 | 100 | 100 | 100 |
| **LEGEND:** | | | | | | | | | | | | | | | | | | | | |
| **—** **Data not available** | | | | | | | | | | | | | | | | | | | | |
| **Low rates (<10%) of CMC enrollment** | | | | | | | | | | | | | | | | | | | | |
| **Low concern** | | | | | | | | | | | | | | | | | | | | |
| **Did not meet criteria** | | | | | | | | | | | | | | | | | | | | |

**Abbreviations:** CMC = comprehensive managed care; FFS = fee-for-service; SD = standard deviation

**Notes:** Measure was defined as the percentage of inpatient claims with a non-missing value in the fill date field. State-years were identified as low concern if data for CMC enrollees were comparable to (i.e. within 2 standard deviations of) the national FFS population in the year.

# **Figure A12. CMC Results for Percentage of Pharmacy Claims with a National Drug Code**

| **STATE** | | **2001** | **2002** | **2003** | **2004** | **2005** | **2006** | **2007** | **2008** | **2009** | **2010** | **2011** | **2012** | **2013** | **2014** | **2015** | **2016** | **2017** | **2018** | **2019** |
| --- | --- | --- | --- | --- | --- | --- | --- | --- | --- | --- | --- | --- | --- | --- | --- | --- | --- | --- | --- | --- |
| **AK** | |  |  |  |  |  |  |  |  |  |  |  |  |  |  |  |  |  |  |  |
| **AL** | |  |  |  |  |  |  |  |  |  |  |  |  |  |  |  |  |  |  |  |
| **AR** | |  |  |  |  |  |  |  |  |  |  |  |  |  |  |  |  |  |  |  |
| **AZ** | | — | — | — | — | — | — | — | — | — | — | — | — | — |  |  |  |  |  |  |
| **CA** | |  |  |  |  |  |  |  |  |  |  |  |  |  |  |  |  |  |  |  |
| **CO** | |  |  |  |  |  |  |  |  |  |  |  |  |  |  |  |  |  |  |  |
| **CT** | |  |  |  |  |  |  |  |  |  |  |  |  |  |  |  |  |  |  |  |
| **DC** | | — | — | — | — | — | — | — | — | — | — | — | — | — |  |  |  |  |  |  |
| **DE** | | — | — | — | — | — | — | — | — | — | — | — | — | — |  |  |  |  |  |  |
| **FL** | |  |  |  |  |  |  |  |  |  |  |  |  |  |  |  |  |  |  |  |
| **GA** | |  |  |  |  |  |  |  |  |  |  |  |  |  |  |  |  |  |  |  |
| **HI** | |  |  |  |  |  |  |  |  |  |  |  |  |  |  |  |  |  |  |  |
| **IA** | |  |  |  |  |  |  |  |  |  |  |  |  |  |  |  |  |  |  |  |
| **ID** | |  |  |  |  |  |  |  |  |  |  |  |  |  |  |  |  |  |  |  |
| **IL** | |  |  |  |  |  |  |  |  |  |  |  |  |  |  |  |  |  |  |  |
| **IN** | |  |  |  |  |  |  |  |  |  |  |  |  |  |  |  |  |  |  |  |
| **KS** | |  |  |  |  |  |  |  |  |  |  |  |  |  |  |  |  |  |  |  |
| **KY** | |  |  |  |  |  |  |  |  |  |  |  |  |  |  |  |  |  |  |  |
| **LA** | |  |  |  |  |  |  |  |  |  |  |  |  |  |  |  |  |  |  |  |
| **MA** | |  |  |  |  |  |  |  |  |  |  |  |  |  |  |  |  |  |  |  |
| **MD** | |  |  |  |  |  |  |  |  |  |  |  |  |  |  |  |  |  |  |  |
| **ME** | |  |  |  |  |  |  |  |  |  |  |  |  |  |  |  |  |  |  |  |
| **MI** | |  |  |  |  |  |  |  |  |  |  |  |  |  |  |  |  |  |  |  |
| **MN** | |  |  |  |  |  |  |  |  |  |  |  |  |  |  |  |  |  |  |  |
| **MO** | |  |  |  |  |  |  |  |  |  |  |  |  |  |  |  |  |  |  |  |
| **MS** | |  |  |  |  |  |  |  |  |  |  |  |  |  |  |  |  |  |  |  |
| **MT** | |  |  |  |  |  |  |  |  |  |  |  |  |  |  |  |  |  |  |  |
| **NC** | |  |  |  |  |  |  |  |  |  |  |  |  |  |  |  |  |  |  |  |
| **ND** | |  |  |  |  |  |  |  |  |  |  |  |  |  |  |  |  |  |  |  |
| **NE** | |  |  |  |  |  |  |  |  |  |  |  |  |  |  |  |  |  |  |  |
| **NH** | |  |  |  |  |  |  |  |  |  |  |  |  |  |  |  |  |  |  |  |
| **NJ** | |  |  |  |  |  |  |  |  |  |  |  |  |  |  |  |  |  |  |  |
| **NM** | |  |  |  |  |  |  |  |  |  |  |  |  |  |  |  |  |  |  |  |
| **NV** | | — | — | — | — | — | — | — | — | — | — | — | — | — |  |  |  |  |  |  |
| **NY** | |  |  |  |  |  |  |  |  |  |  |  |  |  |  |  |  |  |  |  |
| **OH** | |  |  |  |  |  |  |  |  |  |  |  |  |  |  |  |  |  |  |  |
| **OK** | |  |  |  |  |  |  |  |  |  |  |  |  |  |  |  |  |  |  |  |
| **OR** | | — | — | — | — | — | — | — | — | — | — | — | — | — |  |  |  |  |  |  |
| **PA** | |  |  |  |  |  |  |  |  |  |  |  |  |  |  |  |  |  |  |  |
| **RI** | | — | — | — | — | — | — | — | — | — | — | — | — | — |  |  |  |  |  |  |
| **SC** | |  |  |  |  |  |  |  |  |  |  |  |  |  |  |  |  |  |  |  |
| **SD** | |  |  |  |  |  |  |  |  |  |  |  |  |  |  |  |  |  |  |  |
| **TN** | |  |  |  |  |  |  |  |  |  |  |  |  |  |  |  |  |  |  |  |
| **TX** | |  |  |  |  |  |  |  |  |  |  |  |  |  |  |  |  |  |  |  |
| **UT** | |  |  |  |  |  |  |  |  |  |  |  |  |  |  |  |  |  |  |  |
| **VA** | |  |  |  |  |  |  |  |  |  |  |  |  |  |  |  |  |  |  |  |
| **VT** | |  |  |  |  |  |  |  |  |  |  |  |  |  |  |  |  |  |  |  |
| **WA** | |  |  |  |  |  |  |  |  |  |  |  |  |  |  |  |  |  |  |  |
| **WI** | |  |  |  |  |  |  |  |  |  |  |  |  |  |  |  |  |  |  |  |
| **WV** | |  |  |  |  |  |  |  |  |  |  |  |  |  |  |  |  |  |  |  |
| **WY** | |  |  |  |  |  |  |  |  |  |  |  |  |  |  |  |  |  |  |  |
| **FFS** | **Mean** | 100 | 100 | 100 | 100 | 100 | 100 | 100 | 100 | 100 | 100 | 100 | 100 | 100 | 99.9 | 99.7 | 97.6 | 99.6 | 98.5 | 99.8 |
|  | **SD** | 0 | 0 | 0 | 0 | 0 | 0 | 0.1 | 0.1 | 0.1 | 0 | 0 | 0.1 | 0.1 | 0.3 | 0.8 | 14.7 | 1.2 | 7.4 | 0.6 |
| **CMC** | **Min** | 100 | 100 | 100 | 99.9 | 99.9 | 100 | 100 | 100 | 99.9 | 99.9 | 99.9 | 99.9 | 99.8 | 99.7 | 99.6 | 86.8 | 99.5 | 99.4 | 99.6 |
|  | **Max** | 100 | 100 | 100 | 100 | 100 | 100 | 100 | 100 | 100 | 100 | 100 | 100 | 100 | 100 | 100 | 100 | 100 | 100 | 100 |
| **LEGEND:** | | | | | | | | | | | | | | | | | | | | |
| **—** **Data not available** | | | | | | | | | | | | | | | | | | | | |
| **Low rates (<10%) of CMC enrollment** | | | | | | | | | | | | | | | | | | | | |
| **Low concern** | | | | | | | | | | | | | | | | | | | | |
| **Did not meet criteria** | | | | | | | | | | | | | | | | | | | | |

**Abbreviations:** CMC = comprehensive managed care; FFS = fee-for-service; SD = standard deviation

**Notes:** Measure was defined as the percentage of pharmacy claims with a non-missing value in the National Drug Code (NDC) field, considering fields with all zeros, nines, dots, or spaces as missing. State-years were identified as low concern if data for CMC enrollees were comparable to (i.e. within 2 standard deviations of) the national FFS population in the year.

# **Figure A13. CMC Results for Percentage of Pharmacy Claims with Days Supplied**

| **STATE** | | **2001** | **2002** | **2003** | **2004** | **2005** | **2006** | **2007** | **2008** | **2009** | **2010** | **2011** | **2012** | **2013** | **2014** | **2015** | **2016** | **2017** | **2018** | **2019** |
| --- | --- | --- | --- | --- | --- | --- | --- | --- | --- | --- | --- | --- | --- | --- | --- | --- | --- | --- | --- | --- |
| **AK** | |  |  |  |  |  |  |  |  |  |  |  |  |  |  |  |  |  |  |  |
| **AL** | |  |  |  |  |  |  |  |  |  |  |  |  |  |  |  |  |  |  |  |
| **AR** | |  |  |  |  |  |  |  |  |  |  |  |  |  |  |  |  |  |  |  |
| **AZ** | | — | — | — | — | — | — | — | — | — | — | — | — | — |  |  |  |  |  |  |
| **CA** | |  |  |  |  |  |  |  |  |  |  |  |  |  |  |  |  |  |  |  |
| **CO** | |  |  |  |  |  |  |  |  |  |  |  |  |  |  |  |  |  |  |  |
| **CT** | |  |  |  |  |  |  |  |  |  |  |  |  |  |  |  |  |  |  |  |
| **DC** | | — | — | — | — | — | — | — | — | — | — | — | — | — |  |  |  |  |  |  |
| **DE** | | — | — | — | — | — | — | — | — | — | — | — | — | — |  |  |  |  |  |  |
| **FL** | |  |  |  |  |  |  |  |  |  |  |  |  |  |  |  |  |  |  |  |
| **GA** | |  |  |  |  |  |  |  |  |  |  |  |  |  |  |  |  |  |  |  |
| **HI** | |  |  |  |  |  |  |  |  |  |  |  |  |  |  |  |  |  |  |  |
| **IA** | |  |  |  |  |  |  |  |  |  |  |  |  |  |  |  |  |  |  |  |
| **ID** | |  |  |  |  |  |  |  |  |  |  |  |  |  |  |  |  |  |  |  |
| **IL** | |  |  |  |  |  |  |  |  |  |  |  |  |  |  |  |  |  |  |  |
| **IN** | |  |  |  |  |  |  |  |  |  |  |  |  |  |  |  |  |  |  |  |
| **KS** | |  |  |  |  |  |  |  |  |  |  |  |  |  |  |  |  |  |  |  |
| **KY** | |  |  |  |  |  |  |  |  |  |  |  |  |  |  |  |  |  |  |  |
| **LA** | |  |  |  |  |  |  |  |  |  |  |  |  |  |  |  |  |  |  |  |
| **MA** | |  |  |  |  |  |  |  |  |  |  |  |  |  |  |  |  |  |  |  |
| **MD** | |  |  |  |  |  |  |  |  |  |  |  |  |  |  |  |  |  |  |  |
| **ME** | |  |  |  |  |  |  |  |  |  |  |  |  |  |  |  |  |  |  |  |
| **MI** | |  |  |  |  |  |  |  |  |  |  |  |  |  |  |  |  |  |  |  |
| **MN** | |  |  |  |  |  |  |  |  |  |  |  |  |  |  |  |  |  |  |  |
| **MO** | |  |  |  |  |  |  |  |  |  |  |  |  |  |  |  |  |  |  |  |
| **MS** | |  |  |  |  |  |  |  |  |  |  |  |  |  |  |  |  |  |  |  |
| **MT** | |  |  |  |  |  |  |  |  |  |  |  |  |  |  |  |  |  |  |  |
| **NC** | |  |  |  |  |  |  |  |  |  |  |  |  |  |  |  |  |  |  |  |
| **ND** | |  |  |  |  |  |  |  |  |  |  |  |  |  |  |  |  |  |  |  |
| **NE** | |  |  |  |  |  |  |  |  |  |  |  |  |  |  |  |  |  |  |  |
| **NH** | |  |  |  |  |  |  |  |  |  |  |  |  |  |  |  |  |  |  |  |
| **NJ** | |  |  |  |  |  |  |  |  |  |  |  |  |  |  |  |  |  |  |  |
| **NM** | |  |  |  |  |  |  |  |  |  |  |  |  |  |  |  |  |  |  |  |
| **NV** | | — | — | — | — | — | — | — | — | — | — | — | — | — |  |  |  |  |  |  |
| **NY** | |  |  |  |  |  |  |  |  |  |  |  |  |  |  |  |  |  |  |  |
| **OH** | |  |  |  |  |  |  |  |  |  |  |  |  |  |  |  |  |  |  |  |
| **OK** | |  |  |  |  |  |  |  |  |  |  |  |  |  |  |  |  |  |  |  |
| **OR** | | — | — | — | — | — | — | — | — | — | — | — | — | — |  |  |  |  |  |  |
| **PA** | |  |  |  |  |  |  |  |  |  |  |  |  |  |  |  |  |  |  |  |
| **RI** | | — | — | — | — | — | — | — | — | — | — | — | — | — |  |  |  |  |  |  |
| **SC** | |  |  |  |  |  |  |  |  |  |  |  |  |  |  |  |  |  |  |  |
| **SD** | |  |  |  |  |  |  |  |  |  |  |  |  |  |  |  |  |  |  |  |
| **TN** | |  |  |  |  |  |  |  |  |  |  |  |  |  |  |  |  |  |  |  |
| **TX** | |  |  |  |  |  |  |  |  |  |  |  |  |  |  |  |  |  |  |  |
| **UT** | |  |  |  |  |  |  |  |  |  |  |  |  |  |  |  |  |  |  |  |
| **VA** | |  |  |  |  |  |  |  |  |  |  |  |  |  |  |  |  |  |  |  |
| **VT** | |  |  |  |  |  |  |  |  |  |  |  |  |  |  |  |  |  |  |  |
| **WA** | |  |  |  |  |  |  |  |  |  |  |  |  |  |  |  |  |  |  |  |
| **WI** | |  |  |  |  |  |  |  |  |  |  |  |  |  |  |  |  |  |  |  |
| **WV** | |  |  |  |  |  |  |  |  |  |  |  |  |  |  |  |  |  |  |  |
| **WY** | |  |  |  |  |  |  |  |  |  |  |  |  |  |  |  |  |  |  |  |
| **FFS** | **Mean** | 100 | 100 | 100 | 100 | 100 | 100 | 100 | 100 | 100 | 100 | 100 | 100 | 100 | 100 | 99.7 | 99.6 | 99.7 | 99.2 | 99.7 |
|  | **SD** | 0 | 0 | 0 | 0 | 0 | 0 | 0 | 0 | 0 | 0 | 0 | 0 | 0 | 0.2 | 1.3 | 2.0 | 1.7 | 5.2 | 1.4 |
| **CMC** | **Min** | 100 | 100 | 100 | 100 | 100 | 100 | 100 | 100 | 100 | 100 | 100 | 100 | 100 | 99.7 | 98.9 | 99.4 | 99.8 | 99.8 | 99.8 |
|  | **Max** | 100 | 100 | 100 | 100 | 100 | 100 | 100 | 100 | 100 | 100 | 100 | 100 | 100 | 100 | 100 | 100 | 100 | 100 | 100 |
| **LEGEND:** | | | | | | | | | | | | | | | | | | | | |
| **—** **Data not available** | | | | | | | | | | | | | | | | | | | | |
| **Low rates (<10%) of CMC enrollment** | | | | | | | | | | | | | | | | | | | | |
| **Low concern** | | | | | | | | | | | | | | | | | | | | |
| **Did not meet criteria** | | | | | | | | | | | | | | | | | | | | |

**Abbreviations:** CMC = comprehensive managed care; FFS = fee-for-service; SD = standard deviation

**Notes:** Measure was defined as the percentage of pharmacy claims with a non-missing value in the days supplied field, considering fields with all dots or spaces as missing. State-years were identified as low concern if data for CMC enrollees were comparable to (i.e. within 2 standard deviations of) the national FFS population in the year.

# **Figure A14. CMC Results for Percentage of Pharmacy Claims with Quantity Dispensed**

| **STATE** | | **2001** | **2002** | **2003** | **2004** | **2005** | **2006** | **2007** | **2008** | **2009** | **2010** | **2011** | **2012** | **2013** | **2014** | **2015** | **2016** | **2017** | **2018** | **2019** |
| --- | --- | --- | --- | --- | --- | --- | --- | --- | --- | --- | --- | --- | --- | --- | --- | --- | --- | --- | --- | --- |
| **AK** | |  |  |  |  |  |  |  |  |  |  |  |  |  |  |  |  |  |  |  |
| **AL** | |  |  |  |  |  |  |  |  |  |  |  |  |  |  |  |  |  |  |  |
| **AR** | |  |  |  |  |  |  |  |  |  |  |  |  |  |  |  |  |  |  |  |
| **AZ** | | — | — | — | — | — | — | — | — | — | — | — | — | — |  |  |  |  |  |  |
| **CA** | |  |  |  |  |  |  |  |  |  |  |  |  |  |  |  |  |  |  |  |
| **CO** | |  |  |  |  |  |  |  |  |  |  |  |  |  |  |  |  |  |  |  |
| **CT** | |  |  |  |  |  |  |  |  |  |  |  |  |  |  |  |  |  |  |  |
| **DC** | | — | — | — | — | — | — | — | — | — | — | — | — | — |  |  |  |  |  |  |
| **DE** | | — | — | — | — | — | — | — | — | — | — | — | — | — |  |  |  |  |  |  |
| **FL** | |  |  |  |  |  |  |  |  |  |  |  |  |  |  |  |  |  |  |  |
| **GA** | |  |  |  |  |  |  |  |  |  |  |  |  |  |  |  |  |  |  |  |
| **HI** | |  |  |  |  |  |  |  |  |  |  |  |  |  |  |  |  |  |  |  |
| **IA** | |  |  |  |  |  |  |  |  |  |  |  |  |  |  |  |  |  |  |  |
| **ID** | |  |  |  |  |  |  |  |  |  |  |  |  |  |  |  |  |  |  |  |
| **IL** | |  |  |  |  |  |  |  |  |  |  |  |  |  |  |  |  |  |  |  |
| **IN** | |  |  |  |  |  |  |  |  |  |  |  |  |  |  |  |  |  |  |  |
| **KS** | |  |  |  |  |  |  |  |  |  |  |  |  |  |  |  |  |  |  |  |
| **KY** | |  |  |  |  |  |  |  |  |  |  |  |  |  |  |  |  |  |  |  |
| **LA** | |  |  |  |  |  |  |  |  |  |  |  |  |  |  |  |  |  |  |  |
| **MA** | |  |  |  |  |  |  |  |  |  |  |  |  |  |  |  |  |  |  |  |
| **MD** | |  |  |  |  |  |  |  |  |  |  |  |  |  |  |  |  |  |  |  |
| **ME** | |  |  |  |  |  |  |  |  |  |  |  |  |  |  |  |  |  |  |  |
| **MI** | |  |  |  |  |  |  |  |  |  |  |  |  |  |  |  |  |  |  |  |
| **MN** | |  |  |  |  |  |  |  |  |  |  |  |  |  |  |  |  |  |  |  |
| **MO** | |  |  |  |  |  |  |  |  |  |  |  |  |  |  |  |  |  |  |  |
| **MS** | |  |  |  |  |  |  |  |  |  |  |  |  |  |  |  |  |  |  |  |
| **MT** | |  |  |  |  |  |  |  |  |  |  |  |  |  |  |  |  |  |  |  |
| **NC** | |  |  |  |  |  |  |  |  |  |  |  |  |  |  |  |  |  |  |  |
| **ND** | |  |  |  |  |  |  |  |  |  |  |  |  |  |  |  |  |  |  |  |
| **NE** | |  |  |  |  |  |  |  |  |  |  |  |  |  |  |  |  |  |  |  |
| **NH** | |  |  |  |  |  |  |  |  |  |  |  |  |  |  |  |  |  |  |  |
| **NJ** | |  |  |  |  |  |  |  |  |  |  |  |  |  |  |  |  |  |  |  |
| **NM** | |  |  |  |  |  |  |  |  |  |  |  |  |  |  |  |  |  |  |  |
| **NV** | | — | — | — | — | — | — | — | — | — | — | — | — | — |  |  |  |  |  |  |
| **NY** | |  |  |  |  |  |  |  |  |  |  |  |  |  |  |  |  |  |  |  |
| **OH** | |  |  |  |  |  |  |  |  |  |  |  |  |  |  |  |  |  |  |  |
| **OK** | |  |  |  |  |  |  |  |  |  |  |  |  |  |  |  |  |  |  |  |
| **OR** | | — | — | — | — | — | — | — | — | — | — | — | — | — |  |  |  |  |  |  |
| **PA** | |  |  |  |  |  |  |  |  |  |  |  |  |  |  |  |  |  |  |  |
| **RI** | | — | — | — | — | — | — | — | — | — | — | — | — | — |  |  |  |  |  |  |
| **SC** | |  |  |  |  |  |  |  |  |  |  |  |  |  |  |  |  |  |  |  |
| **SD** | |  |  |  |  |  |  |  |  |  |  |  |  |  |  |  |  |  |  |  |
| **TN** | |  |  |  |  |  |  |  |  |  |  |  |  |  |  |  |  |  |  |  |
| **TX** | |  |  |  |  |  |  |  |  |  |  |  |  |  |  |  |  |  |  |  |
| **UT** | |  |  |  |  |  |  |  |  |  |  |  |  |  |  |  |  |  |  |  |
| **VA** | |  |  |  |  |  |  |  |  |  |  |  |  |  |  |  |  |  |  |  |
| **VT** | |  |  |  |  |  |  |  |  |  |  |  |  |  |  |  |  |  |  |  |
| **WA** | |  |  |  |  |  |  |  |  |  |  |  |  |  |  |  |  |  |  |  |
| **WI** | |  |  |  |  |  |  |  |  |  |  |  |  |  |  |  |  |  |  |  |
| **WV** | |  |  |  |  |  |  |  |  |  |  |  |  |  |  |  |  |  |  |  |
| **WY** | |  |  |  |  |  |  |  |  |  |  |  |  |  |  |  |  |  |  |  |
| **FFS** | **Mean** | 100 | 100 | 100 | 100 | 100 | 100 | 100 | 100 | 100 | 100 | 100 | 100 | 100 | 100 | 99.8 | 97.7 | 98.1 | 99.6 | 99.9 |
|  | **SD** | 0 | 0 | 0 | 0 | 0 | 0 | 0 | 0 | 0 | 0 | 0 | 0 | 0 | 0.2 | 0.7 | 14.7 | 11.7 | 2.2 | 0.6 |
| **CMC** | **Min** | 100 | 100 | 100 | 100 | 100 | 100 | 100 | 100 | 100 | 100 | 100 | 100 | 100 | 99.7 | 98.9 | 94.1 | 99.7 | 99.7 | 99.7 |
|  | **Max** | 100 | 100 | 100 | 100 | 100 | 100 | 100 | 100 | 100 | 100 | 100 | 100 | 100 | 100 | 100 | 100 | 100 | 100 | 100 |
| **LEGEND:** | | | | | | | | | | | | | | | | | | | | |
| **—** **Data not available** | | | | | | | | | | | | | | | | | | | | |
| **Low rates (<10%) of CMC enrollment** | | | | | | | | | | | | | | | | | | | | |
| **Low concern** | | | | | | | | | | | | | | | | | | | | |
| **Did not meet criteria** | | | | | | | | | | | | | | | | | | | | |

**Abbreviations:** CMC = comprehensive managed care; FFS = fee-for-service; SD = standard deviation

**Notes:** Measure was defined as the percentage of pharmacy claims with a non-missing value in the quantity dispensed field, considering fields with all dots or spaces as missing. State-years were identified as low concern if data for CMC enrollees were comparable to (i.e. within 2 standard deviations of) the national FFS population in the year.

# **Table A5. Proportion of All State-Years with Low-Concern CMC Data, 2001-2019 (n=891)**

|  | **Inpatient** | | **Outpatient** | | **Prescription** | | **Overall** | |
| --- | --- | --- | --- | --- | --- | --- | --- | --- |
|  | **N** | **%** | **N** | **%** | **N** | **%** | **N** | **%** |
| **Completeness** | 781 | 87.7 | 818 | 91.8 | 810 | 90.9 | 758 | 85.1 |
| Any | 835 | 93.7 | 821 | 92.1 | 826 | 92.7 | — | — |
| Mean | 800 | 89.8 | 853 | 95.7 | 822 | 92.3 | — | — |
| **Quality** | 800 | 89.8 | 862 | 96.7 | 879 | 98.7 | 768 | 86.2 |
| Diagnosis | 867 | 97.3 | 872 | 97.9 | — | — | — | — |
| Procedure | 819 | 91.9 | 880 | 98.8 | — | — | — | — |
| Fill date | — | — | — | — | 884 | 99.2 | — | — |
| NDC | — | — | — | — | 881 | 98.9 | — | — |
| Days supplied | — | — | — | — | 882 | 99.0 | — | — |
| Quantity dispensed | — | — | — | — | 883 | 99.1 | — | — |

**Abbreviations:** CMC = comprehensive managed care

**Notes:** All state-years included those with <10% CMC enrollment. The proportion of state-years with low-concern CMC data among state-years with ≥10% CMC enrollment is shown in Table 1. Completeness measures for state-years with ≥10% CMC enrollment were defined as: (1) the percentage of enrollees with any inpatient, outpatient, or prescription drug claim and (2) the mean number of inpatient, outpatient, and prescription drug claims per enrollees with ≥1 claim. Quality measures for state-years with ≥10% CMC enrollment were defined as: (1) the percentage of inpatient/outpatient claims with a primary diagnosis and procedure code and (2) the percentage of prescription drug claims with a fill date, National Drug Code (NDC), days supplied, and quantity dispensed.

# **Figure A15. Proportion of All States with Low-Concern CMC Data by Year, 2001-2019**

**Abbreviations:** CMC = comprehensive managed care

**Notes**: All states included those with <10% CMC enrollment. The proportion of analyzed states (≥10% CMC enrollment) with low-concern CMC data is shown in Figure 2. Mean data completeness and quality were calculated as the weighted average across the study period, where weighting reflects the number of state-years included in analyses.
